# Unraveling the Shared Genetic Architecture and Polygenic Overlap Between Loneliness, Major Depressive Disorder, and Sleep-Related Traits

**DOI:** 10.3390/biomedicines13123101

**Published:** 2025-12-16

**Authors:** Zainab Rehman, Abdul Aziz Khan, Jun Ye, Xianda Ma, Yifang Kuang, Ziying Wang, Zhaohui Lan, Qian Zhao, Jiarun Yang, Xu Zhang, Sanbing Shen, Weidong Li

**Affiliations:** 1 Key Laboratory for the Genetics of Development and Neuropsychiatric Disorders, Institute of Psychology and Behavioral Sciences, Bio-X Institutes, Shanghai Jiao Tong University, Shanghai 200240, China; 2 Brain Health and Brain Technology Center, Global Institute of Future Technology, Shanghai Jiao Tong University, Shanghai 200240, China; 3 Regenerative Medicine Institute (REMEDI), School of Medicine, College of Medicine, Nursing and Health Sciences, University of Galway, H91 TK33 Galway, Ireland

**Keywords:** loneliness, major depressive disorder, sleep, gene expression, genetic risk factors, genome-wide association study, single nucleotide polymorphism, polygenic overlap, linkage disequilibrium, transcriptome-wide association study

## Abstract

**Background**: Loneliness (LON) is a heritable psychosocial trait that frequently co-occurs with major depressive disorder (MDD) and sleep traits. Despite known genetic contributions, the shared genetic architecture and molecular mechanisms underlying their co-occurrence remain largely unknown. This study aimed to uncover novel genetic risk loci and cross-trait gene expression effects. **Methods**: Large-scale genome-wide association study (GWAS) datasets were analyzed using the causal mixture model (MiXeR) to estimate polygenicity and shared genetic architecture. Genetic correlation analyses were performed using linkage disequilibrium score regression (LDSC) and local analysis of [co]variant annotation (LAVA). Conditional and conjunctional FDR methods further identified single nucleotide polymorphisms (SNPs). FUMA was used for gene mapping and annotation, and transcriptome-wide association studies (TWAS) assessed cross-trait gene expression effects. **Results**: Analyses revealed extensive polygenic overlap between LON, MDD, and sleep-related traits, with concordant and discordant effects. Several novel loci were identified, and cross-trait gene expression effects were observed in multiple brain-expressed genes, including *WNT3*, *ARHGAP27*, *PLEKHM1*, and *FOXP2*. These findings provide insight into the shared genetic architecture and relevance of these traits. **Conclusions**: This study demonstrates a significant shared polygenic architecture among LON, MDD, and sleep traits, providing new biological insights. It advances our understanding of cross-trait genetic mechanisms and identifies potential targets for future research, offering broader implications for trait co-occurrence.

## 1. Introduction

Loneliness (LON) has emerged as a growing public health concern, increasingly recognized as a determinant of both physical and mental health outcomes. Defined by limited or unsatisfactory social interactions, loneliness not only reflects a subjective sense of social disconnection but also represents a major risk factor for a wide range of somatic and psychiatric conditions, including major depressive disorder (MDD), schizophrenia, and autism spectrum disorders [1,2,3,4,5,6,7]. Clinical and epidemiological studies have indicated that individuals experiencing LON are more likely to exhibit elevated stress levels, limited social support, and increased vulnerability to mood and anxiety disorders [6,8,9]. Among these associated conditions, MDD is particularly significant, as it frequently co-occurs with loneliness and amplifies its detrimental effects [10,11,12].

MDD is one of the leading causes of disability worldwide, affecting more than 264 million people. It is characterized by persistent sadness, reduced motivation, impaired cognitive and social functioning, and an increased risk of suicide [13,14]. Both MDD and LON have a strong heritable component, as twin and family-based studies reporting heritability estimates of approximately 37% for MDD [15,16,17] and 40–50% for loneliness [12,18,19,20]. These genetic findings highlight the need to investigate the common biological mechanisms that may account for the frequent co-occurrence of loneliness and depression.

Despite, sleep-related traits have also been increasingly recognized as a crucial factor contributing to the bidirectional relationship between loneliness and depression. Sleep disturbances, such as insomnia (INS), chronotype (CHR), and altered sleep duration (SD), are commonly reported among individuals experiencing LON and those with depressive symptoms [21,22]. Insomnia, which affects up to 30% of the global population, is one of the most prevalent sleep disorders and has been consistently associated with both LON and depressive symptoms [23,24,25]. Similarly, disruptions in CHR, which reflect individual differences in sleep–wake preferences, have been linked to an increased risk of depression and anxiety [26,27]. Short or poor-quality sleep duration is also associated with increased depressive symptom burden and reduced emotional resilience [23,28]. Genetic factors play an important role in these sleep-related traits, with heritability estimates ranging between 31% and 55% [29,30,31], suggesting that shared genetic mechanisms may contribute to their frequent co-occurrence with MDD and LON.

Recent genome-wide association studies (GWAS) have provided important insights into the polygenic nature of these traits. Large-scale GWAS have identified multiple risk loci, including 15 for LON [19], 44 for MDD [32], 202 for INS [33], 351 for CHR [34], and 78 for SD [35]. These discoveries emphasize the extensive genetic architecture underlying each phenotype. Importantly, evidence of genetic correlations among these traits suggests overlapping biological mechanisms: genetic studies have reported significant correlations between LON and MDD [36], as well as between MDD and sleep phenotypes such as INS and CHR [34,37,38]. Observational and bidirectional Mendelian randomization studies have further demonstrated that LON can increase vulnerability to poor sleep quality and depression, reinforcing the notion of shared genetic liability [35,38].

While these studies have greatly advanced our understanding, several gaps remain. Traditional genome-wide genetic correlation analyses, such as linkage disequilibrium score regression (LDSC) [39], offer useful estimates of overall genetic overlap but are limited in their ability to dissect the detailed structure of shared genetic effects. Specifically, they cannot distinguish between concordant and discordant effect directions, nor can they localize the genetic signals to specific regions or genes [40,41,42]. Consequently, the extent and nature of the polygenic overlap among LON, MDD, and sleep traits remain incompletely characterized. Addressing these limitations requires approaches that move beyond global correlation estimates toward models capable of identifying shared loci, gene-level associations, and relevant biological pathways.

Although the comorbidity among LON, MDD, and sleep disturbances has been well established at the phenotypic level, the shared genetic mechanisms that may underlie this co-occurrence are still poorly understood. Most prior work has either examined pairwise genetic correlations or focused on single-trait analyses, leaving the broader genetic architecture linking these interrelated traits largely unresolved. Understanding the genetic overlap between LON, MDD, and sleep traits is crucial, not only for clarifying their biological basis but also for identifying potential targets for intervention. Shared genetic risk factors could point to convergent neurobiological pathways, such as stress reactivity, circadian rhythm regulation, or neurotransmitter signaling, that influence multiple aspects of mental health.

In this study, we aimed to provide a comprehensive analysis of the shared genetic basis underlying LON, MDD, and sleep-related traits, including INS, CHR, and SD. By integrating large-scale GWAS datasets, we examined both common and trait-specific components of their genetic architecture. Our approach focused on identifying overlapping genetic variants and genes that may contribute to neurobiological processes involved in emotion regulation and sleep–wake function. Beyond clarifying the degree of polygenic overlap, this study offers a more mechanistic view of the shared genetic foundations of psychiatric comorbidity.

Altogether, these findings allow for a more nuanced understanding of the genetic relationships linking LON, MDD, and sleep traits. Clarifying their shared polygenic architecture improves insight into the biological processes influencing vulnerability to mental health disorders and supports the development of more targeted approaches in psychiatric research and care.

## 2. Materials and Methods

This study was designed to identify the shared and distinct genetic components contributing to LON, MDD, and sleep-related traits. We used summary statistics from large genome-wide association studies (GWAS) based on European samples to ensure the data were comparable. The analysis followed a stepwise framework combining several statistical approaches suitable to detect shared genetic signals and polygenicity among these traits. First, we used MiXeR to describe the overall polygenic structure and estimate the number of variants influencing each trait [40,43]. We then applied LDSC [32,34,39] and LAVA [42] to assess both global and regional genetic correlations. To improve the discovery of common loci, we employed the conditional and conjunctional false discovery rate (condFDR and conjFDR) approaches [44,45,46]. These results were later annotated and interpreted using functional mapping and transcriptome-wide association analyses [47,48]. Together, these complementary approaches provided a robust framework to evaluate the shared genetic basis of loneliness, depression, and sleep traits.

### 2.1. Study Sample for GWAS Analysis

**Figure 1:**
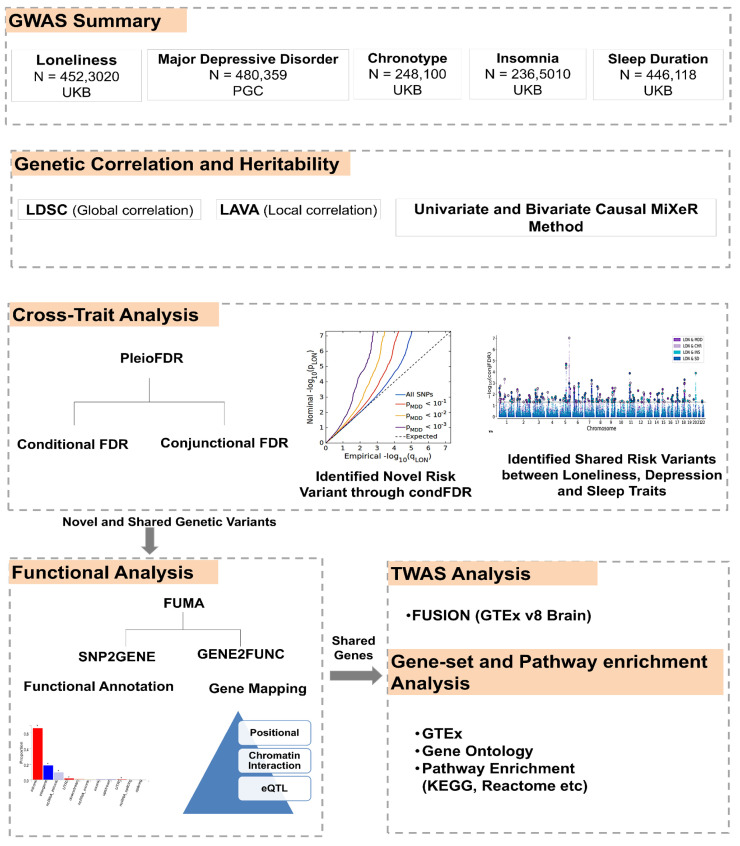
Flowchart of the study design. Summary of datasets and analytical workflow used to examine shared genetic architecture between loneliness (LON), major depressive disorder (MDD), and sleep-related traits (INS, CHR, and SD) using GWAS data and integrative statistical and functional analyses.

The GWAS dataset for LON was obtained from the UK Biobank, including 452,302 individuals, based on three self-reported questions, such as a feeling of loneliness, social interaction frequency, and the presence of someone to confide in [19]. The GWAS dataset for MDD was accessed from the Psychiatric Genomic Consortium (PGC). This dataset encompassed 135,458 MDD cases and 344,901 control subjects [32]. Further, GWAS datasets for sleep traits such as INS, CHR and SD were obtained from a UK biobank study. The INS sample dataset included 593,724 cases and 1,771,286 controls [33]. CHR encompassed a dataset of 248,100 participants, including 120,478 cases and 127,622 controls [34], and the SD data were obtained from 446,118 European individuals [35]. All GWAS datasets were obtained from the European population. More information on the method details for GWAS summary statistics of all phenotypic traits is provided in Supplementary Materials file S1 (Table S1) and in the original GWAS publications [19,32,33,34,35]. An overview of the study design, including dataset sources and analytical workflow, is shown in Figure 1.

### 2.2. Statistical Analysis

#### 2.2.1. Conditional Q-Q Plot

The quantitative assessment of true associations and statistical enrichments was derived through analysis of the summary statistics. We created a conditional Q-Q plot for the primary trait (LON) by filtering SNPs when conditioned on secondary traits (MDD, INS, CHR, SD) and vice versa to visualize pleiotropic enrichment among these traits. In this analysis, we adopted a more comprehensive approach by integrating polygenic effects with sub-threshold *p*-values (*p* > 5 × 10^−8^) to elucidate their potential effects. The observed *p*-values presented a progressive leftward deflection away from the expected distribution and, under the global null hypothesis, showed pleiotropic effects. This leftward deflection suggests a non-random increase in the SNPs related to the primary trait as their association strength with the secondary trait increases (at *p* values < 1, 0.1, 0.01, 0.001) [44,47,49].

#### 2.2.2. Univariate and Bivariate Gaussian Causal MiXeR Method

We utilized causal MiXeR (version 1.3) methods [40] (https://github.com/precimed/mixer (accessed on 15 March 2025)) [50]; with GWAS summary data to investigate the polygenic architecture and genetic overlap among LON, MDD, and sleep-related traits (CHR, INS, and SD). MiXeR estimates the total number of shared and trait-specific causal variants, which were summarized in Venn diagrams showing the proportion of unique and overlapping SNPs along with standard deviations derived from multiple independent iterations. Univariate analyses were conducted to estimate SNP-based heritability, polygenicity, discoverability, and the number of causal variants explaining 90% of SNP heritability [51]. For cross-trait analyses, we applied bivariate MiXeR methods [40] to quantify the genetic overlap between each trait pair, including the number of shared causal variants irrespective of effect direction, and calculate the Dice coefficient (i.e., the proportion of shared variants out of the total influencing both traits). Enrichment patterns were examined using conditional Q-Q plots. Additionally, MiXeR estimates the genome-wide SNP correlations (rg) and effect size correlations within the shared genetic component (rgs). Detailed descriptions of the MiXeR methods can be found in the Supplementary Materials (Supplementary Material File S1).

#### 2.2.3. Effect Direction, Genetic Heritability, and Genetic Correlation Using LDSC and LAVA

The effect direction of shared genomic risk loci of MDD, LON, and sleep traits (CHR, INS, and SD) was calculated by analyzing the z-scores of lead SNPs. We used the Linkage Disequilibrium Regression Score (LDSC) [39] for the estimation of genetic heritability and genetic correlation. By utilizing the LAVA [Local Analysis of [co]Variant Association] framework [42], we further investigated local genetic correlation and heritability between all phenotypes of interest, which divides the genome into approximately 2495 semi-independent regions, each around 1 Mb in scale. To ensure the validity of the analysis, only the regions with significant estimated SNP heritability (*p* < 0.05/2495) were included to estimate local genetic correlations across all trait pairs.

#### 2.2.4. Conditional/Conjunctional False Discovery Rate (CondFDR/ConjFDR) Method

We then utilized the conditional FDR (condFDR) approach [44,45] to boost the identification of SNPs associated with LON, MDD, and sleep traits (INS, CHR, and SD). The condFDR method builds on the standard FDR by integrating GWAS summary datasets of primary and secondary traits to modify its significance criteria. The SNP ranking was based on standard FDR or *p*-value, resulting in an identical sequence. However, if there is a genetic association between the primary and secondary phenotypes, the condFDR rearranges SNP strata, resulting in a different order than obtained from *p*-values alone. By reversing the role of both traits, we obtained the reverse condFDR values. The conjFDR approach was applied [44] to determine the overlapping genetic risk variants between LON, MDD, and other secondary phenotypes. We also applied the conjFDR (an extension of condFDR), which is the posterior probability that an SNP shows no association with either one or both phenotypes, where its *p*-values for both traits are as small or smaller than the observed one. We determined the maximum condFDR for each SNP by repeatedly applying the condFDR approach to both phenotypes, reversing their roles to ensure cautious estimation of conjFDR.

In condFDR, we set a threshold of less than 0.01 and 0.05 in conjFDR for each pairwise comparison [44]. All analyses were conducted following the exclusion of single nucleotide polymorphisms (SNPs) located within two specific genomic regions: the extended major histocompatibility complex (MHC) found on chromosome 6 [hg19: 25,119,106-33,854,733] and the 8p23.1 on chromosome 8 [hg19: 7,242,715-12,483,982], to reduce LD confounding effects that show influence on cond/conjFDR estimation. Further information on these methods can be found in the original publication [44], related studies [19,32,33,34,35], and the additional information (Supplementary Materials File S1).

#### 2.2.5. Defining Genomic Loci

The Functional Mapping and Annotation (FUMA) protocol facilitates the identification of independent risk loci [47]. All identified candidate SNPs were used to define distinct genomic risk loci at linkage disequilibrium LD (r^2^ ≥ 0.6) to one of the leading and independent SNPs in the locus. Some of these variants were specifically selected as lead SNPs at LD r^2^ < 0.1. The most significant genetic variants at LD r^2^ < 0.6 with condFDR below 0.01 and conjFDR under 0.05 were recognized as independent SNPs [44]. All physically overlapping SNPs selected as lead SNPs were merged less than 250 kb apart. LD data were sourced from the reference panel provided by the 1000 Genomes Project [52]. The genetic variants for loneliness, MDD, or sleep-related phenotypes not found in the original GWAS, previous condFDR/conjFDR studies, or GWAS catalog were considered novel.

### 2.3. Functional Annotation

The candidate SNPs were functionally annotated within loci by using ANNOVAR (version 2025Mar02) [53] in FUMA (version 1.3.7) [47]. The Combined Annotation-Dependent Depletion (CADD) score [54] predicts deleteriousness, RegulomeDB (RDB) scores [55] assess the regulatory functions, while Chromatin States [56,57] indicate regulatory influences on the SNP locus. Next, we utilized three gene mapping approaches provided by FUMA to assign independent candidate SNPs within shared loci to brain-expressed genes. Moreover, we used two different strategies to check the novelty of genetic variants. We first compared our data with already published condFDR/conjFDR results related to our traits, then used the NHGRI-EBI catalog [58] for the identification of loci overlapping with previously reported GWAS studies. For gene set and pathway analysis, Gene Ontology (GO) [59] was analyzed on all the genes nearest to the common and shared variants identified through condFDR/conjFDR analysis by using the FUMA tool [47], whereas the GTEx [60] was used to describe likely regulatory lead SNPs within a biological context.

### 2.4. Transcriptome-Wide Association Study (TWAS)

TWAS was conducted using the FUSION package [48] to identify associations between genetically regulated gene expression and each phenotype (LON, MDD, INS, CHR, SD). The expression of trait-associated genes was evaluated in a tissue-specific manner using 13 GTEx brain tissues [60], and overlapping genes across traits were identified to highlight candidates underlying shared genetic architecture. Bonferroni correction *p* < 0.05/*n*) was applied to determine the significant associations.

### 2.5. Software and Packages

All statistical analyses were conducted using R (version 4.3.1), Python (version 2.7 and 3.8), and MATLAB R2023a. Genome-wide genetic correlations were estimated with LDSC software (v1.0.1) [39], while local genetic correlations were examined using LAVA (version 0.1.5) [42]. Polygenicity and shared genetic architecture were assessed with the causal MiXeR method (v1.3) [40]. Shared loci were identified using the conditional and conjunctional false discovery rate (condFDR/conjFDR) framework [44]. Gene mapping and functional annotation were performed with FUMA (v1.3.7) [47]. Transcriptome-wide association studies (TWAS) were conducted using FUSION software which is maintained as a collection of R scripts and pre-compiled binaries on GitHub (gusevlab/fusion_twas) [48]. R packages (version 4.3.1) and Python (version 3.8) were applied where relevant for data preprocessing, statistical modeling, and visualization.

### 2.6. Ethical Statement

This study used publicly available GWAS summary statistics only. No individual-level data were used. Therefore, no new ethical approval or participant consent was required. Ethical approvals for each original GWAS can be found in their respective publications.

## 3. Results

### 3.1. Cross-Trait Enrichment Pattern Analysis

**Figure 2:**
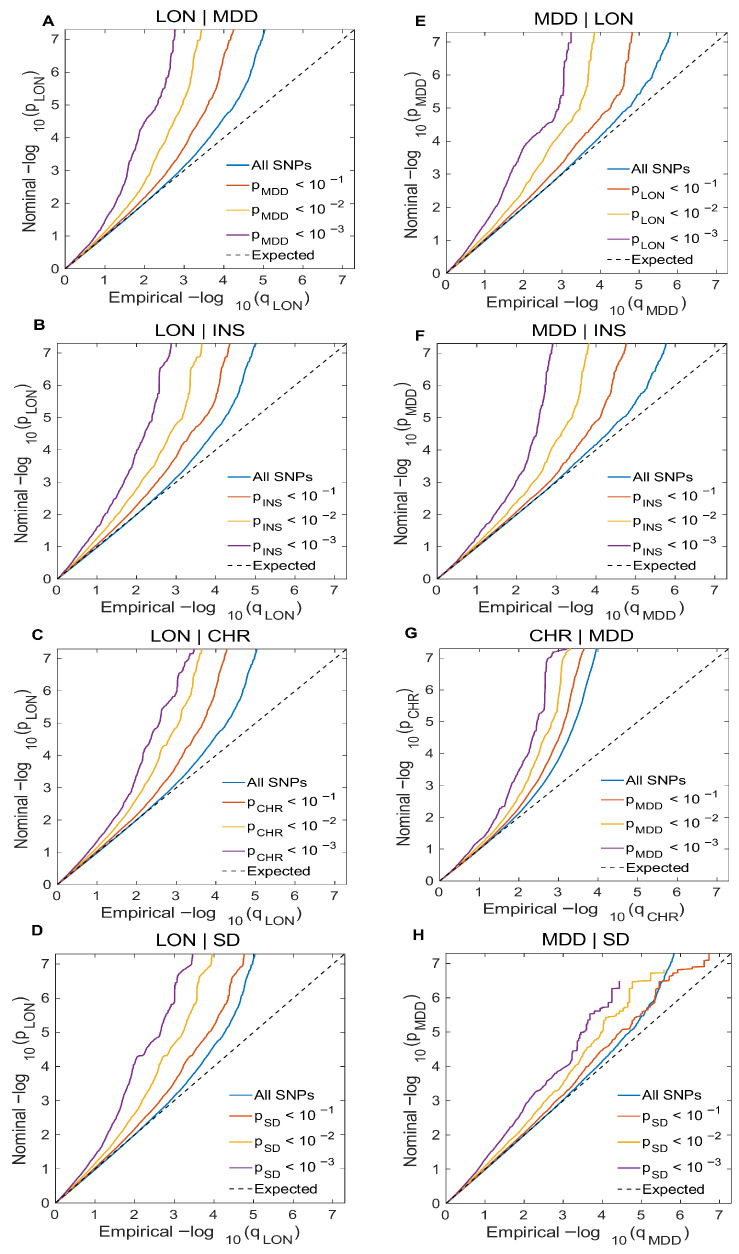
Genetic overlap between LON, MDD, and sleep traits. Conditional Q-Q plots display polygenic enrichment among LON, MDD and sleep traits, by plotting the nominal *p*-values against the inflation-adjusted empirical −log10 (*p*) values for the phenotypes under investigation following standard GWAS criteria of *p* < 5 × 10^−8^. Significant association with the conditional trait is represented by -log10 *p*-values cut-offs (*p* < 0.10, 0.01, 0.001. The SNPs are represented by a blue line, with the dashed line indicating the null hypothesis. (**A**–**D**) LON is conditioned on MDD, INS, CHR, and SD, respectively. (**E**–**H**) MDD is conditioned on LON, INS, CHR, and SD.

Conditional Q-Q plots revealed a significant pattern of association for LON when conditioned on MDD, INS, CHR, SD, and vice versa. Similarly, an enrichment pattern for MDD is observed when conditioned on INS, CHR, and SD (Figure 2). This polygenic enrichment was observed in both directions, reflecting a significant genetic overlap. The SNPs are represented by the blue line within the primary traits (LON and MDD), independent of their associations with the other traits. Meanwhile, the SNP subsets for association *p*-values with significant increases in the conditional trait are represented by red, yellow, and purple lines in Figure 2. The consistent leftward deflection of these subsets shows a significant polygenic overlap between loneliness, major depression, and sleep-related phenotypes, suggesting a shared genetic architecture between these phenotypes (Figure 2). The visual representation of reverse conditional Q-Q plots is shown in Supplementary Material File S1: Figure S1.

### 3.2. Genetic Overlap Quantification and Polygenicity Between LON, MDD, and Sleep Traits Using Univariate and Bivariate Causal MiXeR Method

**Figure 3:**
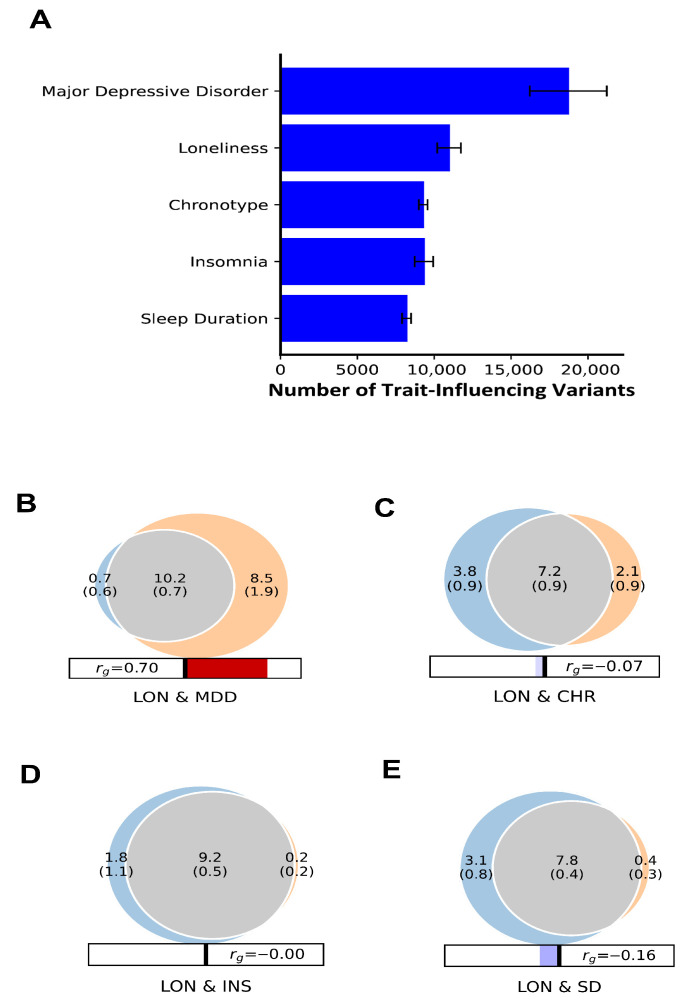
Univariate and Bivariate MiXeR Analysis. (**A**) Univariate analysis estimated the polygenicity of LON, MDD, and sleep-related traits by a number of trait-influencing causal variants accounting for 90% of the heritability. (**B**–**E**) Venn diagrams illustrating shared (gray) and unique trait-influencing causal variants between (**B**) LON (blue) and MDD, (**C**) LON and CHR, (**D**) LON and INS, and (**E**) LON and SD (orange). The rg refers to the genome-wide significant correlation (directional scale: positive, red; negative, blue). The size of the circle corresponds to the polygenicity of each trait, with large circles representing the higher polygenicity.

The univariate MiXeR analysis identified high polygenicity for LON with 10.9K trait-influencing causal variants, accounting for 90% heritability. The number of trait-influencing variants for MDD (18.7K), CHR (9.2K), INS (9.3K), and SD (8.2K) indicates a substantial genetic contribution of all these phenotypes (Figure 3). The supplementary result section found the MiXeR Q-Q plot for observed versus expected *p*-values and log-likelihood plots for shared variants among these phenotypes (Supplementary Material File S1: Figure S2).

The Bivariate MiXeR analysis found that LON shared an estimated 10.2K variants out of 19.4K at 90% heritability with MDD with a Dice coefficient (DC) of 69%, that indicating a significant genetic overlap, as illustrated by the Venn diagram (Figure 3A). For other trait pairs, LON and CHR shared 7.2K of 13.1K variants (DC = 71%), LON and INS shared 9.2K of 11.2K variants (DC = 90%), and LON and SD shared 7.8K of 11.3K variants (DC = 82%). These findings reveal considerable polygenic overlap despite a wide range of genetic correlations (rg = −0.07 to 0.7) (Figure 3B–D). The highest genetic correlation was observed between LON and MDD (rg = 0.7), largely consistent with previous findings (Table 1). In terms of congruency, the strongest concordance in effect directions was observed between LON and MDD, followed by LON and INS. Moderate concordant effects were noted between LON and CHR and between LON and SD, indicating mixed effect directions across these trait pairs.

The MiXeR result estimates the robust positive Akaike information criterion (AIC) scores for LON and INS (72.31) and LON and CHR (29.47), suggesting better model fits and significant shared genetic components. Whereas, for LON and MDD, the model fit shows a suboptimal negative AIC score. In the context of the MiXeR model, a negative AIC indicates that the best-fitting bivariate model may not improve upon the simplest model, suggesting that the shared genetic component between LON and MDD could be smaller than estimated or that there is limited statistical power for this analysis (see Supplementary Material File S2: Table S5).

### 3.3. Genetic Correlation and Effect Directions

Using LD score regression analysis, we found a significant positive genetic correlation between LON and MDD (*rg* = 0.067) and an inverse genetic correlation with SD (*rg* = −0.15), but not significant for INS and CHR. Furthermore, a strong genetic correlation between MDD and INS was identified. In contrast, a moderate correlation was observed between MDD and CHR (*p* < 0.004, *rg* = −0.068) and MDD and SD (*p* < 0.01, *rg* = −0.08) (Supplementary Material File S2: Table S68). By considering the direction of allelic effects of shared lead SNPs at conjFDR < 0.05, we also found consistent effect direction between LON and MDD (93.5%), whereas LON showed 28.5% same effect direction with INS, 33.3% with CHR, and 24% with sleep duration (see Supplementary Material File S1 for additional details).

### 3.4. Local Genetic Correlation Through LAVA Analysis

**Figure 4:**
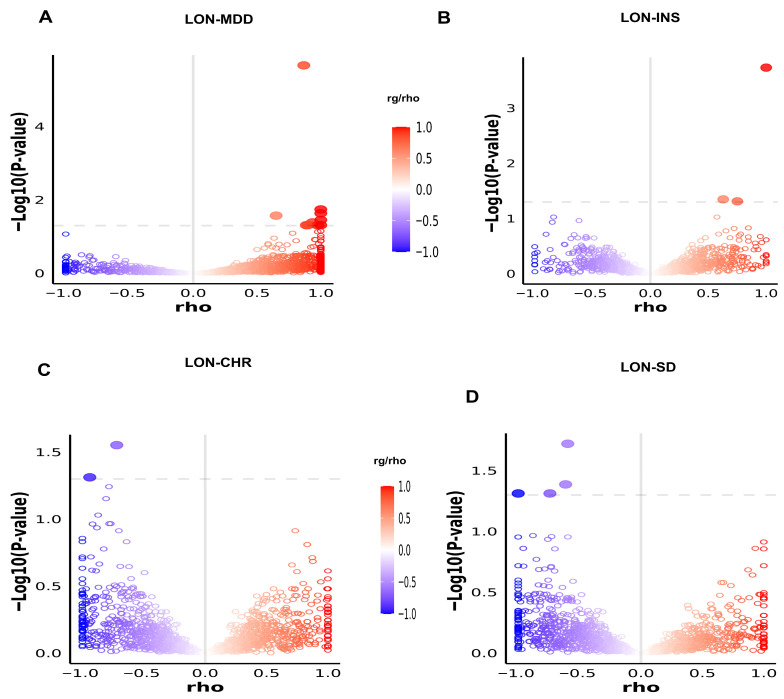
Volcano plot of LAVA local genetic correlation analysis (rg/rho with −log10 *p* values for each locus (**A**–**D**). The filled circles showed significant loci after multiple testing corrections. The blue outline circles represent a negative genetic correlation, and the red outline circles with a positive genetic correlation. The gray vertical line in the center separates loci with positive versus negative correlation.

The overall genetic correlation between two traits may be negligible when investigated at the genome-wide level. To determine this, we estimated regional genetic correlation by applying the LAVA [42]. Loci having significant local genetic correlation (*p* < 0.05/2495) were included in bivariate analysis. This method estimated a mixture of positive and negative local genetic correlations. LAVA analysis reveals that loci have a significant positive local genetic correlation between LON and MDD (*n* = 10), INS *(n* = 3), and a negative genetic correlation between LON and CHR (*n* = 4), SD (*n* = 2) after multiple testing correction (Figure 4; Supplementary Material File S1: Table S4). These findings are consistent with MiXeR-estimated mixed-effect directions between all phenotypes.

### 3.5. Association of Genomic Loci with LON, MDD and Sleep-Related Traits

At condFDR < 0.01, we identified 52, 43, 38, and 45 LD-independent genomic risk loci related to LON conditioned on MDD, INS, CHR, and SD. Whereas 10, 21, 9, and 27 loci were identified as associated with MDD when conditioned on all sleep traits and LON (Table 2 and Supplementary Material File S2: Tables S6–S13). We also employed a conditional Manhattan plot to visualize the localization of genetic factors (Supplementary Material File S1: Figure S4). We further found 178 loci associated with LON, of which 113 newly identified loci were absent from previously reported original GWAS, previous cond/conjFDR analyses, or the GWAS catalog (Supplementary Material File S2: Table S34). Additionally, 29 genomic loci were related to MDD, all of which had not been reported previously in the original genome-wide association studies or GWAS catalog (Supplementary Material File S2: Table S35). In the reversed condFDR analysis, 70 loci were associated with INS, 536 loci with CHR, and 259 with SD, conditional on major depression (condFDR < 0.01). Among these, 19 INS loci, 192 CHR loci, and 124 SD risk loci were absent from previously reported original GWAS, prior condFDR analysis, or the GWAS catalog (Supplementary Material File S2: Tables S14–S19).

Several distinct genomic loci were identified as overlapping between LON, MDD, and sleep traits at conjFDR < 0.05 (Supplementary Material File S1: Table S2). LON revealed 62 overlapping risk loci with MDD, 56 with INS, 54 with CHR, and 62 with SD (Supplementary Material File S2: Tables S20–S23). In total, 234 loci were jointly associated, of which 211 loci were novel for LON (Supplementary Material File S2: Table S39). Furthermore, MDD shared 19, 17, and 15 genomic risk loci with INS, CHR and SD, respectively (Supplementary Material File S1: Table S2). In total, 51 were unique to MDD and sleep traits, resulting in the discovery of 22 novel risk factors for MDD that were not included in previous GWAS catalog records (Supplementary Material File S2: Table S40).

**Figure 5:**
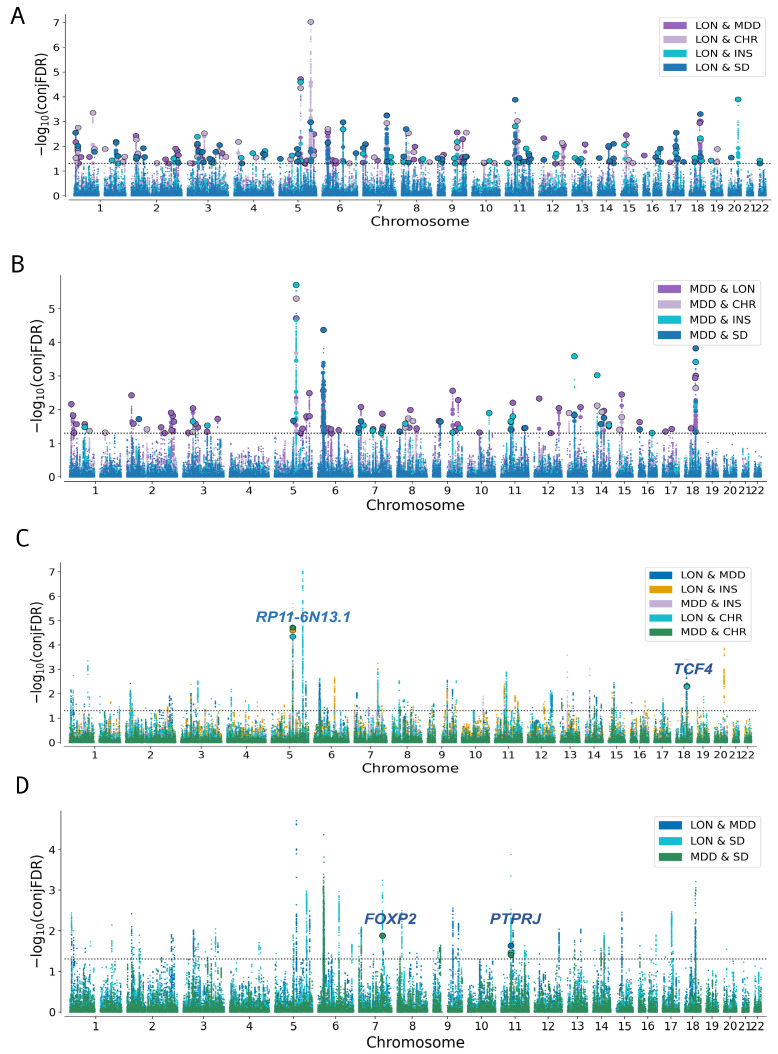
Shared and common genetic variants among LON, MDD, and sleep-related traits at conjFDR < 0.05. This figure presents Manhattan plots illustrating shared variants among (**A**) LON with MDD, CHR, INS, and SD, (**B**) MDD with LON, INS, CHR, and SD, (**C**,**D**) common variants among all phenotypes. The *y*-axis illustrates −log10 transformed conjFDR values for the SNPs, whereas the *x*-axis shows chromosomal positions. Black circles surrounding the larger data points represent the significant SNPs found in each LD block. The dashed horizontal line denotes the significant threshold to identify shared and common associations when conjFDR is less than 0.05.

Further, combined conjFDR analyses show common variants between LON, MDD, INS (2), CHR (2), and SD (2) (Supplementary Material File S1: Table S3 and Supplementary Material File S2: Tables S27–S33). The conjFDR Manhattan plots for all traits also uncovered independent lead SNPs marked with black circles (Figure 5 and Supplementary Material File S1: Figure S3).

### 3.6. Functional Annotations of Genetic Loci

**Figure 6:**
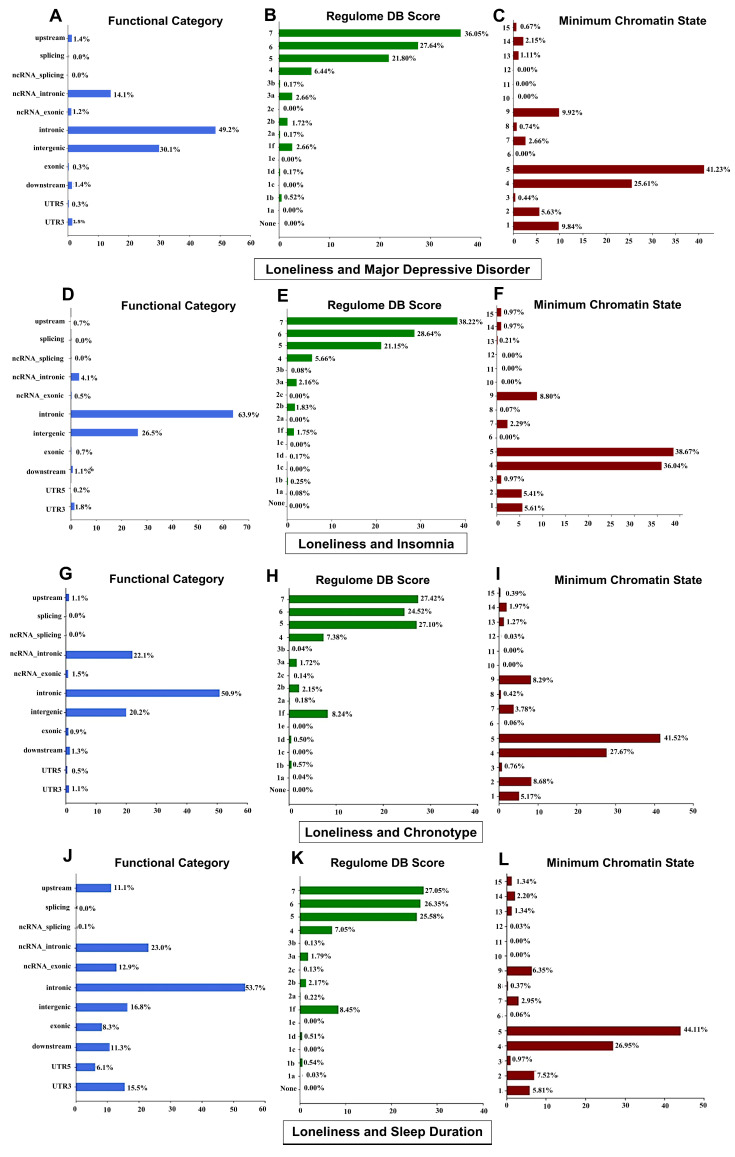
(**A**–**L**) The functional annotation distribution, RegulomeDB Score, and Chromatin interaction mapping of all SNPs within the common genetic risk variants between loneliness, major depression, and sleep traits at conjFDR < 0.10.

We utilized FUMA to functionally annotate all SNPs exhibiting linkage disequilibrium (*r*^2^  ≥  0.6) significantly associated with an independent SNP at conjFDR threshold below 0.1 within the loci related to LON, MDD, and sleep-related traits. Most variants jointly associated with LON, MDD, and sleep traits are intronic and intergenic. Among them, several loci showed high deleteriousness (Figure 6; Supplementary Material File S1: Figure S5 and Supplementary Material File S2: Tables S27–S33).

### 3.7. Gene Mapping and Gene-Set Enrichment Analysis

Next, we applied three independent gene-mapping strategies to assign candidate SNPs to brain-expressed genes (Supplementary Material File S2: Tables S50–S56). Multiple SNPs were associated with genes through positional, chromatin interaction, and eQTL mapping across various human brain tissues, mostly the adult cortex.

We further identified the linked genes through gene set analysis and found that 176 genes are mapped between LON and MDD, 212 between LON and INS, 366 between LON and CHR, and 379 between LON and SD. Additionally, we found 88 genes mapped between MDD and INS, 43 between MDD and CHR and 165 genes between MDD and SD (Supplementary Material File S2: Tables S54–S56). Among the identified genes, 120 were mapped using two of the three distinct gene mapping methods (Supplementary Material File S2: Table S67).

Additionally, we identified 37 unique and most credible genes associated with shared loci mapped through all gene mapping strategies (see Table 3 and Supplementary Material File S2: Table S57). Out of these genes, we found five credible genes jointly related to LON, MDD, CHR and SD. Moreover, we found eight credible genes were linked to LON, CHR and SD, suggesting potential pleiotropic effects, while the remaining genes were associated with two related phenotypes (Table 3).

We also identified an overrepresentation of these mapped genes in 51 significant Gene Ontology (GO) terms for LON, MDD, and sleep traits associated with several biological, molecular and cellular components (“structural constituent of chromatin”, “protein heterodimerization activity”, “nucleosome organization”, “DNA packaging complex”, “odorant binding” and “olfactory receptor activity”, etc.) through gene set enrichment analysis (GSEA) (Supplementary Material File S2: Table S58).

### 3.8. TWAS Identified Brain-Expressed Genes with Cross-Trait Overlap

We also identified significant TWAS genes (Bonferroni-corrected *p* < 0.05/number of genes per GTEx brain panel) (Supplementary Material File S2: Tables S69–S74), which emphasized tissue-specific effects of gene expression on each trait. Several genes mapped by SNP-based positional, chromatin, and eQTL approaches were also significant in TWAS, supporting their functional relevance. Notably, genes such as *WNT3, ARHGAP27, PLEKHM1*, and *FOXP2* were implicated across multiple brain tissues for LON, MDD, and sleep-related traits, supporting shared genetic mechanisms (Supplementary Material File S1: Figure S6).

### 3.9. Pathway Analysis

Gene set pathway analysis revealed several overrepresented pathways associated with LON, MDD, and sleep traits after excluding MHC regions [Supplementary Material File S2: Tables S59–S64]. The most common and shared pathways were “systemic lupus erythematosus”, “signaling by *Wnt*”, “Notch signaling”, “signaling by nuclear receptors”, “reactome_signaling_by_rho_gtpases_miro_gtpases_and_rhobtb3”, “olfactory signaling pathway”, “ESR mediated signaling”, etc. See Supplementary Material File S2: Tables S59–S64 for all overrepresented pathways.

Overall, the analyses show that LON, MDD, and sleep-related traits share a substantial number of genetic variants, while some variants are unique to each trait. This indicates both common and distinct genetic contributions across these phenotypes that may underlie their shared and specific biological mechanisms.

## 4. Discussion

To our knowledge, this is one of the first studies to examine LON, MDD, and several sleep traits together within the same genetic framework. By integrating large-scale GWAS datasets and applying complementary statistical approaches, we identified extensive shared and distinct polygenic components across LON, MDD, and the sleep-related traits (INS, CHR and SD). The findings emphasize that the frequent clinical co-occurrence of these phenotypes is not incidental but rather reflects shared biological factors that influence emotional regulation, social behavior, and circadian processes [61,62,63]. This work thus advances our understanding of the complex genetic relationships contributing to psychiatric and behavioral comorbidities and offers a foundation for future research into their molecular and clinical implications. This reinforces the view LON is not only a psychosocial experience but also has a biological foundation that overlaps strongly with depression and sleep regulation [10,35].

Through the cond/conjFDR framework, we detected hundreds of novel loci associated with LON, MDD, and sleep-related traits. This approach greatly enhanced discovery power by leveraging the polygenic enrichment shared across phenotypes. Notably, 234 loci were jointly associated across all traits, and many of these had not been previously reported. These results suggest that pleiotropic variants, rather than disorder-specific ones, may play a major role in driving psychiatric comorbidity. Our findings suggest that different genetic loci may modulate distinct neurobiological pathways, contributing variably to the pathophysiology of psychiatric and neurological traits. Highly pleiotropic variants instead of disorder-specific variants could be the key contributors to the risk of multiple mental health issues and associated phenotypes. This aligns with the fact that most genetic risk variants associated with mental disorders are predominantly found in regulatory regions rather than coding sequences. As a result, allelic differences may consequently influence neurobiological pathways in varying directions, leading to phenotypic differences through common pathways [41,64,65]. Local genetic correlation analyses further revealed a mixture of agonistic and antagonistic effects across different genomic regions [66,67,68], supporting the view that distinct loci may influence convergent or divergent neurobiological pathways.

Importantly, by applying MiXeR and LAVA, we were able to show that the overlap extends beyond simple genetic correlations, capturing more detailed patterns of shared and opposing effects. The simultaneous occurrence of both concordant and discordant directions of effect suggests that shared loci may have complex effects on these traits, sometimes increasing risk across multiple conditions and at other times exerting opposing effects [66,69]. For instance, non-linear and even counterintuitive clinical associations between LON and sleep-related traits could be mediated by mixed effect directions, given that non-linear and counterintuitive clinical patterns can be witnessed in some individuals even with shared genetic risk factors. This suggests that certain genetic variants may simultaneously increase susceptibility to LON while predisposing individuals to either shorter or longer sleep duration, depending on the specific genomic context, environmental exposures, or other biological compensatory processes [37,70]. The importance of identifying such complicated genetic interactions is critical for understanding the heterogeneity of the clinical manifestations, as well as the interventions based on the specific genetic risks could be designed. Such patterns emphasize the biological heterogeneity of psychiatric and sleep-related phenotypes and suggest that different genetic pathways may converge on similar clinical outcomes [44,69].

Furthermore, functional annotation and gene-set enrichment analyses offered insight into the molecular mechanisms linking these traits. We observed enrichment in various biological mechanisms such as chromatin organization, brain functions, olfactory signaling, protein heterodimerization, immune response, *Wnt* and *Notch* signaling, and circadian regulation, etc. These findings converge with growing evidence that alterations in synaptic plasticity, neuroinflammatory responses, and transcriptional control contribute to the pathophysiology of both depression and sleep disruption. Particularly noteworthy was the enrichment of olfactory signaling pathways, which have been implicated in sensory processing, memory formation, and affective regulation [71]. Disruptions in olfactory function have been observed in patients with depression and anxiety disorders [72], suggesting a potential sensory-affective interface through which genetic variation could influence mood and social perception. Similarly, *Wnt* and *Notch* signaling pathways, key regulators of neuronal differentiation and plasticity were linked to both depression and circadian rhythm regulation, providing a plausible biological bridge between mood dysregulation and sleep–wake disturbances [73,74,75,76].

Integration of TWAS results further supported the involvement of brain-expressed genes such as *WNT3, ARHGAP27, PLEKHM1*, and *FOXP2* [77,78,79,80]. These genes are statistically implicated in synaptic transmission, axonal growth, and cognitive-emotional regulation, but experimental validation is required to establish causal involvement in the shared genetic basis of LON, MDD, and sleep traits. In particular, *FOXP2*, which has been linked to language, emotion, and social communication, may provide a molecular link between social isolation and affective symptoms. Collectively, these functional findings point to an interconnected network of regulatory genes that jointly influence social behavior, mood, and circadian processes. For instance, genes involved in synaptic signaling or circadian regulation may simultaneously affect sleep timing and emotional stability, whereas genes related to stress reactivity or immune response could diverge in their influence on depression versus sleep regulation. Such observations align with current models of psychiatric genetics that emphasize distributed networks of pleiotropic effects rather than single risk loci.

The broader clinical context is also important. A genetic predisposition amplifies the vulnerability to LON, while limited social support and isolation can exacerbate depressive symptoms. The lack of social interaction in individuals may increase perception of social rejection, which further affects their mental health and causes psychiatric conditions like depression and anxiety, and, in return, causes sleep disturbances [62,63,81,82]. Evidence from previous studies suggested that negative social expectations may increase the risk of psychotic symptoms and impact brain structure and function in the social cognitive areas [83]. Moreover, individuals experiencing chronic LON undergo a high-threat perception towards social interactions, contributing to maladaptive cognitive patterns and adverse social experiences, which can further contribute to sleep disruption [11]. Together, these findings support a model in which shared genetic predisposition interacts with adverse social environments to increase risk for comorbid psychiatric and sleep problems.

From a translational perspective, the identification of shared loci and pathways suggests avenues for integrated prevention and treatment. Shared molecular pathways such as *Wnt* signaling, circadian regulators, and synaptic function could be explored as targets for interventions expected to benefit multiple symptom domains (affective, social, and sleep). Polygenic risk profiles that incorporate pleiotropic loci might aid early identification of individuals at elevated risk for comorbid presentations, allowing targeted psychosocial or behavioral interventions (e.g., sleep stabilization, social support enhancement) that address both proximate and downstream consequences. Importantly, translating these genetic findings into practice will require careful validation studies linking genetic risk to intermediate phenotypes (e.g., neural circuit function, sleep physiology) and to treatment response. Clinical trials that stratify participants by polygenic markers could, in the future, test whether interventions targeting shared mechanisms yield broader benefit across comorbid conditions.

The key strength of our study includes the use of multiple complementary methods, which together provided a multidimensional perspective on genetic sharing across LON, MDD and sleep traits. Using the power of genome-wide correlation methods together with locus-level and functional annotation, we were able to not only recapitulate known associations but also identify novel loci and biological pathways that would otherwise be lost using only traditional GWAS methods. The large GWAS sample sizes further increased statistical power, allowing us to detect subtle polygenic overlap among complex traits. Lastly, the combination of statistical genetics with biological context supports the translational aspect of our results, aligning polygenic risk with brain regions and pathways that are already implicated in mood, cognition, and circadian processing.

Despite these strengths, our study has limitations. First, although condFDR/conjFDR methods effectively improve the power to detect shared genetic loci, they identify associated loci rather than definitively causal variants. Therefore, the specific causal SNPs driving these associations remain to be determined, and some observed loci may reflect mediated pleiotropy or linkage effects rather than direct causal effects. Additionally, the identified risk loci could originate from either common or distinct causal variants or from mediated pleiotropy, in which one phenotype affects another [84,85]. To address this, we complemented condFDR/conjFDR with MiXeR analyses, which estimate shared trait-influencing variants and effect direction concordance, providing additional insight into polygenic overlap, but without definitive locus-level causal inference. Future studies using fine-mapping, Mendelian randomization, and causal inference are required to disentangle causal relationships. Second, we used European ancestry GWAS datasets that limit the broader relevance of our results to diverse populations. Differences in allele frequencies, linkage disequilibrium patterns, and environmental exposures in non-European populations may alter the shared genetic architecture. Future studies in diverse populations are required to replicate these findings and to identify population-specific loci that could inform equitable precision medicine approaches. Third, GWAS only identified common risk variants with small effect sizes, possibly missing rare genetic variants that have a significant influence on our traits of interest. Future sequencing-based studies will be necessary to address this gap.

Also, we found significant polygenic overlap among LON, MDD, and sleep disturbances, but these phenotypes may be influenced by environmental and other lifestyle factors that are not fully accounted for in our findings. Although our analyses focus on genetic architecture, the inclusion of environmental moderators, such as social support, stress exposure, and sleep hygiene, would help contextualize polygenic effects within real-world settings. Finally, while gene-set analysis identified potential biological mechanisms and pathways, further experimental and functional validation is essential to confirm the biological relevance of these genes and mechanisms, and to determine how these genetic factors interact with environmental exposures, lifestyle behaviors, and psychosocial stressors. Moreover, while gene-set and enrichment analyses highlight potential biological pathways, functional validation through experimental models is necessary to establish causal relationships.

Looking forward, future research should aim to investigate whether certain shared variants predispose individuals to a cascade from LON to MDD and sleep dysfunction, or conversely whether persistent sleep disturbance promotes social withdrawal and mood symptoms, would deepen understanding of causal mechanisms. Testing identified variants in model systems and leveraging multi-omics datasets will be critical to move from association to function. Extending analyses to ancestrally diverse populations is also essential to assess generalizability and to discover population-specific loci that may inform equitable precision-medicine strategies.

Taken together, our results indicate that LON, MDD, and sleep disturbances share a deeply interconnected biological basis. The overlapping genetic loci and pathways identified here provide a framework for understanding why these conditions frequently co-occur and how they may influence each other across developmental and environmental contexts. This perspective encourages a shift from studying psychiatric disorders in isolation toward an integrative approach that considers shared vulnerability factors and biological networks. Such insight could inform early identification of at-risk individuals and support the development of personalized preventive and therapeutic strategies targeting common neurobiological mechanisms.

## 5. Conclusions

In conclusion, our study highlights the novel and shared genetic overlap influencing LON, MDD, and sleep traits such as INS, CHR, and SD, uncovering a deeper understanding of their common genetic architecture. The overlapping genetic variants and genes emphasize the importance of neurobiological pathways and molecular processes, which may explain the frequent co-occurrence of these traits. The identification of shared genetic factors highlights specific mechanisms that could provide a basis for developing personalized psychiatric treatment approaches and targeted prevention strategies in psychiatric research and clinical care. Despite these developments, the functional impact of many shared variants remains unclear, which highlights the importance of further investigation that would examine their interplay with environmental factors. Addressing these gaps will be critical to translating genetic findings into clinical practice. Future studies could focus on experimental validation of these key genes and on integrating multi-omics approaches to enhance mechanistic understanding. Overall, this work emphasizes the importance of examining cross-trait genetic relationships to better understand the psychiatric comorbidities and to inform interventions aimed at improving mental health outcomes.

## Figures and Tables

**Table 1:** Bivariate MiXeR analysis across all phenotypes. This table presents the proportion of loneliness (LON) specific trait-influencing variants shared with major depressive disorder (MDD) and sleep traits, including chronotype (CHR), insomnia (INS) and sleep duration (SD), and vice versa. The variants explain 90% of SNP heritability of each trait. s.d: standard deviation; DC: Dice coefficient.

Trait 1	Trait 2	% Proportion of LON Shared Variants with MDD and Sleep Traits	% Proportion of MDD and Sleep Traits Shared Variants with LON	DC Mean (%)	Concordance Mean (s.d)	Genetic Correlation Mean (s.d)
LON	MDD	93.5%	54.5%	69%	0.96 (0.04)	0.70 (0.01)
	CHR	66%	98%	71%	0.46 (0.003)	−0.07 (0.01)
	INS	83.5%	98%	90%	0.49 (0.003)	−0.001 (0.01)
	SD	71.5%	95%	82%	0.44 (0.004)	−0.16 (0.01)

**Table 2:** Novel Risk Variants Shared Between LON, MDD, and Sleep-Associated Phenotypes at CondFDR < 0.01.

Primary Trait |Secondary Trait	Genomic Loci at CondFDR < 0.01	Novel Variants in the Primary Trait	Primary Trait|Secondary Trait	Genomic Loci at CondFDR < 0.01	Novel Variants in Primary Trait
LON|MDD	52	23	MDD|LON	27	15
LON|INS	43	31	INS|LON	35	09
LON|CHR	38	25	CHR|LON	274	102
LON|SD	45	34	SD|LON	136	67
MDD|CHR	10	03	CHR|MDD	262	90
MDD|INS	21	07	INS|MDD	35	10
MDD|SD	09	04	SD|MDD	123	57

Note: LON: Loneliness, MDD: Major depressive disorder, INS: Insomnia, CHR: Chronotype, SD: Sleep duration.

**Table 3:** Gene mapped to Independent SNPs between LON, MDD, and Sleep-related Traits through All Three Gene Mapping Approaches at ConjFDR < 0.05.

Ind. SNP Associated Traits	Credible Mapped Gene[s]	No. of Genes
LON, MDD, CHR, SD	*WNT3, RP11-707O23.5, ARHGAP27, PLEKHM1, AC091132.1*	05
LON, CHR, SD	*STH, KANSL1-AS1, ARL17B, SPPL2C, RP11-259G18.3, RP11-259G18.1, CRHR1-IT1, CRHR1*	08
LON, INS, CHR, SD	*FAM180B*	01
LON, MDD	*CCDC71, KLHDC8B, TCTA ^n^, DAG1 ^n^*	04
LON, INS	*FOXP2, FAM120A ^n^, MED27, SPI1, SLC39A13, PSMC3, FAM180B, MTCH2, CLP1, ZDHHC5, SYT1, CSE1L ^n^, DDX27 ^n^, ZNFX1 ^n^*	14
LON, CHR	*FAM180B, ARHGAP27, MAPT-AS1, MAPT, RP11-669E14.6*	05
LON, SD	*RC3H2, NUP160 ^n^, ARHGAP27, PACRG ^n^*	04

Note: Ind. SNP: Independent single nucleotide polymorphism, LON: Loneliness, MDD: Major Depressive Disorder, INS: Insomnia, CHR: Chronotype, SD: Sleep Duration. ^*n*^ Genes associated with genetic risk loci for related phenotypes.

## Data Availability

The GWAS summary statistics used in the current study were publicly available in the following repositories: Loneliness data from the UK Biobank: https://www.repository.cam.ac.uk/handle/1810/277812; MDD from PGC: https://www.med.unc.edu/pgc/; INS: https://ctg.cncr.nl/software/summary_statistics; CHR and SD: https://sleep.hugeamp.org/downloads.html. FUMA v.1.3.7 was used for functional annotation of SNPs with default parameters: https://fuma.ctglab.nl/. All code used in the current study is available online: condFDR/conjFDR: https://github.com/precimed/pleiofdr; LDSC to estimate genetic heritability and correlation: https://github.com/bulik/ldsc; LAVA to estimate local genetic correlation: https://github.com/josefin-werme/LAVA; MiXeR: https://github.com/precimed/mixer (all links were accessed on 15 December 2024). MATLAB R2023a, Python, and R aided in data preprocessing, imaging, and analysis.

## Supplementary Materials

The following supporting information can be downloaded at: https://www.mdpi.com/article/10.3390/biomedicines13123101/s1, File S1: Supplementary methods and Supplementary results [19,32,33,34,35,39,40,44,45,47,52,54,55,56,69,86,87,88,89,90,91,92]; Figure S1: Genetic overlap of sleep traits with LON and MDD. Figure S2: MiXeR figures show polygenicity among LON|MDD and LON|INS, LON|CHR, LON|SD. Figure S3: Manhattan plots for LON, MDD, CHR, and INS at conjFDR < 0.05. Figure S4: Manhattan plots for LON|MDD and LON|INS, LON|CHR, LON|SD. Figure S5: The functional annotation distribution, RegulomeDB Score, and Chromatin interaction mapping of all SNPs within the common risk variants between MDD and sleep traits at conjFDR < 0.10. Figure S6: Heatmaps for LON, MDD, INS, CHR, and SD showing all TWAS significant genes. Supplementary Table S1: Datasets used in the current study. Supplementary Table S2: Novel risk variants associated with LON, MDD, and sleep traits at conjFDR < 0.05. Supplementary Table S3: Common risk factors shared between LON, MDD, and sleep traits at conjFDR < 0.05. Supplementary Table S4: LAVA—Number of positively and negatively correlated genomic regions between LON, MDD, CHR, SD. Supplementary Table S5: Univariate and Bivariate cross trait analysis of LON, MDD and Sleep Traits [INS, CHR, SD] using Causal MiXeR Method. Supplementary Tables S6–S9: Distinct genomic loci associated with LON at condFDR < 0.01 given association with MDD, INS, CHR, and SD, respectively. Supplementary Tables S10–S13: Distinct genomic loci associated with MDD at condFDR < 0.01 given association with INS, CHR, SD, LON, respectively. Supplementary Tables S14 and S15: Distinct genomic loci associated with INS at condFDR < 0.01 given association with LON and MDD. Supplementary Tables S16 and S17: Distinct genomic loci associated with CHR at condFDR < 0.01 given association with LON and MDD. Supplementary Tables S18 and S19: Distinct genomic loci associated with SD at condFDR < 0.01 given association with LON and MDD. Supplementary Tables S20–S23: Distinct shared genomic loci associated with both LON|MDD and LON|INS, LON|CHR, LON|SD at conjFDR < 0.05. Supplementary Tables S24–S26: Distinct shared genomic loci associated with both MDD|INS, MDD|CHR, and MDD|SD at conjFDR < 0.05. Supplementary Tables S27–S30: All candidate SNPs jointly associated with LON|MDD, LON|INS, LON|CHR, LON|SD having a conjFDR < 0.10 and an r2 ≧ 0.6 with one of the independent significant SNPs. Supplementary Tables S31–S33: All candidate SNPs jointly associated with MDD|INS, MDD|CHR, and MDD|SD having a conjFDR < 0.10 and an r2 ≧ 0.6 with one of the independent significant SNPs. Supplementary Tables S34–S38: Novel variants of LON, MDD, INS, CHR, SD at condFDR < 0.01. Supplementary Table S39 and S40: Novel variants of LON and MDD at conjFDR < 0.05. Supplementary Tables S41 and S42: Genomic loci simultaneously associated with LON, MDD, INS, CHR, SD at conjFDR < 0.05. Supplementary Tables S43–S49: eQTL functionality for all lead SNPs associated with LON|MDD, LON, INS, LON|CHR, LON|SD, MDD|INS. MDD|CHR, MDD|SD at conjFDR < 0.05. Supplementary Tables S50–S56: Genes mapped to lead SNPs in loci associated with LON|MDD, LON|INS, LON|CHR, LON|SD, MDD|INS, MDD|CHR, MDD|SD at conjFDR < 0.05. Supplementary Table S57: Credible genes mapped through all gene mapping strategies for LON, MDD and sleep-related phenotypes at conjFDR < 0.05. Supplementary Table S58: Gene-set analysis of SNPs associated genes conjunctionally associated with Loneliness, Major depression and all Sleep traits at conjFDR < 0.05. Supplementary Tables S59–S65: Gene Pathway Analysis for Lead SNPs within Loci Associated with both LON and MDD, conjFDR < 0.05. Supplementary Table S66: Percentage of CADD scores and number of RegulomeDB scores of SNPs for all traits. Supplementary Table S67: Genes mapped to independent SNPs within shared loci that are jointly associated with loneliness and two related phenotypes. Supplementary Table S68: Genetic correlations of LON and MDD with Sleep Traits using LDSC. Supplementary Tables S69–S73: TWAS results for LON, MDD, INS, CHR, and SD.

## Abbreviations

The following abbreviations are used in this manuscript:

LDSC	Linkage disequilibrium score regression
LAVA	Local Analysis of [co]Variant Association
GWAS	Genome-wide association studies
FUMA	Functional mapping and annotation
TWAS	Transcriptome-wide association studies
CondFDR/ConjFDR	Conditional/conjunctional false discovery rate
MHC	Major histocompatibility complex
GTEx	Genotype-tissue expression
rg	Genetic correlation
LON	Loneliness
MDD	Major depressive disorder
INS	Insomnia
CHR	Chronotype
SD	Sleep duration
AIC	Akaike information criterion
DC	Dice coefficient
GO	Gene ontology
CADD	Combined Annotation-Dependent Depletion
ANNOVAR	ANnotation of VARiants
RDB	Regulome database
LD	Linkage disequilibrium
