# Supplementary material for: Unraveling the Shared Genetic Architecture and Polygenic Overlap Between Loneliness, Major Depressive Disorder, and Sleep-Related Traits
Source: Biomedicines. 2025 Dec 16;13(12):3101. doi: 10.3390/biomedicines13123101 (PMC12730563; doi:10.3390/biomedicines13123101)
Supplement: Supplementary file 1 [file biomedicines-13-03101-s001.zip › Supplementary Materials File S1.pdf]

Supplementary Materials File S1

Unraveling the Shared Genetic Architecture and Polygenic Overlap Between Loneliness, Major Depressive Disorder, and Sleep-Related Traits

List of Content	Page No.
Supplementary methods	2 - 7
Table S1	3
Supplementary results	7 - 8
Table S2 – S4	8 - 9
Supplementary Figure S1 – S6	10 - 15
References	16 - 17

Supplementary Methods

GWAS Sample Participants

The methods for defining and measuring phenotypes in each GWAS sample are documented in the original publications. The GWAS datasets for loneliness (LON) were obtained from the UK Biobank, including 452,302 individuals based on three self-reported questions: feelings of loneliness, social interaction frequency, and the presence of someone to confide in [1]. After quality control, the data was available for 452,302 individuals. All traits were derived from self-reported responses to questions administered via touchscreen at the assessment center. The queries included: (a) "Do you often feel lonely?", where responses of 'yes' were classified as cases and 'no' as controls, (b) A composite variable based on household composition and social visit frequency, with cases defined as individuals living alone who reported never having visitors or any external social contact whereas controls were those not living alone or receiving weekly visits, and (c) A variable assessing social interaction quality, "How often are you able to confide in someone close to you?", with cases indicating 'Never or rarely' and controls 'Almost daily' [1]. Following stringent quality control measures, genomic and phenotypic data from the 452,302 individuals were retained for analysis. Additional details are available in the original publication of GWAS [1].

For major depressive disorder (MDD), the GWAS datasets were obtained from the Psychiatric Genomics Consortium (PGC). The dataset encompassed 135,458 MDD cases and 344,901 control subjects [2]. This study conducted a large-scale genome-wide association meta-analysis, merging data from 29 cohorts, including participants of European ancestry. Eligibility for cases to fulfil international diagnostic criteria (DSM-IV, ICD-9, or ICD-10) for a lifetime MDD diagnosis is determined through structured assessments by clinician checklists, reviews of medical records or trained interviewers [2]. Further information can be found in the original GWAS publication [2].

The GWAS datasets for insomnia (INS) were obtained from the UK Biobank (UKB), significantly expanding the sample size to encompass 593,724 cases and 1,771,286 controls by employing a fixed-effect model implemented through METAL software [3]. This expansion substantially enhanced statistical power, facilitating the identification of 554 risk loci, of which 364 were previously unreported [3]. Insomnia assessment within the UKB cohort employed a single-question methodology dichotomized under established protocols. For additional details, please refer to the original article [3].

The chronotype (CHR) included a dataset of 248,100 participants, including 120,478 cases and 127,622 controls, all possessing at least 97% European ancestry [4]. Participants within the UK Biobank reported their chronotype using a single-question format, including "Definitely a 'morning' person" and "Definitely an 'evening' person," with corresponding codes from 2 to -2 [4]. Quantitative and timing assessments of activity levels and sleep patterns were derived using the GGIR software package, both daily and as averages over the monitoring period. A package in GGIR R version 1.5-12 features an innovative algorithm that identifies sleep patterns without a sleep diary and exhibits minimal bias. This algorithm was further validated through polysomnography in an external cohort. More details are available in the original publication [4].

The sleep duration (SD) summary datasets were obtained from 446,118 European populations from UK Biobank [5]. This study identified 78 genetic loci associated with habitual sleep duration, with significant results at $p < 5 \times 10\text{-}8$, and 43 loci showed significant association at $p < 6 \times 10\text{-}9$. Some of these loci, such as PAX8, VRK2, and FBXL12/UBL5/PIN1, were replicated in the CHARGE study where n = 47,180 and p is less than $6.3 \times 10\text{-}4$, showing consistent effects across 55 signals. Further investigation revealed that 78 loci showed associations with accelerometer-tracked sleep duration, inactivity during

the day, sleep frequency and efficiency, involving a total of 85,499 individuals [5]. To reduce potential biases, 24,533 non-white participants were excluded from the analysis. Participants (n ~ 500,000) reported their sleep duration as short (≤ 6 h), normal (7-8 h), or long (≥ 9 h), with extreme responses removed and missing data handled appropriately. Participants using sleep medication were also excluded. Actigraphy devices (Axivity AX3) were used by 103,711 individuals for up to 7 days, between 2.8-9.7 years after the study initiation, providing additional sleep data. For further details, please see the original article [5].

Table S1: Datasets used in the current study

**Phenotype**	**Resource/pubMedIDlink**	**GWAS summary Statistics**	**Publication**
Loneliness (LON)	**PMID:**29970889	http://doi.org/10.17863/CAM.23511	Day et al., Elucidating the genetic basis of social interaction and isolation. *Nature Communication*, 2018.
Major depressive disorder (MDD)	**PMID:**29700475	10.6084/m9.figshare.21655784	Wray et al., Genome-wide association analyses identify 44 risk variants and refine the genetic architecture of major depression. *Nature Genetics*, 2018.
Insomnia (INS)	**PMID:**35835914	https://ctg.cncr.nl/software/summary_statistics	Watanabe et al., Genome-wide meta-analysis of insomnia prioritizes genes associated with metabolic and psychiatric pathways. *Nature Genetics*, 2022.
Chronotype (CHR)	**PMID:**30696823	http://sleep.hugeamp.org/downloads.html	Jones et al., Genome-wide association analyses of chronotype in 697,828 individuals provides insights into circadian rhythms. *Nature Communications*, 2019.
Sleep duration (SD)	**PMID:**30846698	http://sleep.hugeamp.org/downloads.html	Dashti et al., Genome-wide association study identifies genetic loci for self-reported habitual sleep duration supported by accelerometer-derived estimates. *Nature Communications*, 2019.

Causal MiXeR Method

To assess the polygenic overlap between LON, MDD, and sleep traits including INS, SD and CHR, we utilized the MiXeR method to analyze the GWAS summary statistics for these traits.

Univariate Method

In the univariate analysis, MiXeR models the genetic effect for each SNP, i, as a point-normal mixture, $\beta_i = (1-\pi_1)N(0,0) + \pi_1 N(0, \sigma_\beta^2)$, where π_1 represents the proportion of non-null SNPs (i.e., polygenicity) and σ_β^2 represents the variance in effect sizes of the associated SNPs (i.e., discoverability). LD information and allele frequencies were incorporated to estimate the expected probability distribution of the signed test statistic, $z_j = \delta_j + \epsilon_j = \sqrt{N}\sum_i \sqrt{H_i} r_{ij}\beta_i + \epsilon_j$, where N is the sample size, Hi represents the heterozygosity of the i-th SNP, r_{ij} denotes the allelic correlation between SNPs i and j, and $\epsilon_j \sim \mathcal{N}(0, \sigma_0^2)$ is the residual variance. The number of trait-influencing variants was estimated as $M\pi_1$, where M is the number of SNPs in the reference panel, and SNP-based heritability was computed as $\bar{H}\sigma_\beta^2$, where $\bar{H} = \frac{1}{M}\sum_i H_i = 0.2075$ represents the average heterozygosity across the SNPs in the reference panel [6].

Bivariate Method

For the bivariate analysis, MiXeR models the additive genetic effects as a mixture of four components: null SNPs in both traits (π_0), SNPs affecting only the first trait (π_1), SNPs affecting only the second trait (π_2), and SNPs with non-zero effects on both traits (π_{12}). The variance-covariance matrix of the shared component is represented as $\boldsymbol{\Sigma_{12}} = \begin{bmatrix} \sigma_1^2 & \rho_{12}\sigma_1\sigma_2 \\ \rho_{12}\sigma_1\sigma_2 & \sigma_2^2 \end{bmatrix}$ where ρ_{12} represents the correlation of effect sizes within the shared component and σ_1^2 and σ_2^2 are the discoverability parameters estimated from the univariate analyses of the two traits. Genetic correlation r_g between the traits is calculated as $r_g = \frac{\rho_{12}\pi_{12}}{\sqrt{(\pi_1+\pi_{12})(\pi_2+\pi_{12})}}$, and the Dice coefficient (DC) is computed using the formula DC = $\frac{2\pi_{12}}{\pi_1+\pi_2+2\pi_{12}}$.

Cross-trait analyses were performed using 20 iterations of the univariate and bivariate analysis with 2 million randomly selected SNPs, with a minor allele frequency (MAF) threshold of 5% and pruning at an LD threshold of r^2 = 0.8, yielding approximately 600K input SNPs per iteration. The mean and standard deviation estimates for each parameter were calculated from the 20 iterations. Model performance was assessed using Akaike information criterion (AIC), where ($\mathrm{AIC} = 2\mathrm{k} - 2\ln(\mathrm{L})$), with k being the number of free parameters in the model and L being the likelihood function. The AIC was used to evaluate whether the input GWAS summary statistics had sufficient power to distinguish the fitted model from an infinitesimal model, which assumes that all variants are non-null. In bivariate analysis, the AIC was computed for both the full model and reduced models where the polygenic overlap parameter π_{12} was constrained to either minimal or maximal values, with ($\pi_{12}^{min} = r_g\sqrt{\pi_1^u\,\pi_2^u}$ and $\pi_{12}^{max} = min(\pi_1^u, \pi_2^u)$), respectively. Positive ΔAIC values suggest that the GWAS data have sufficient power to differentiate between the fitted polygenic overlap model from the constrained ones. The polygenic overlap results are presented in Venn diagrams that depict shared and unique polygenic components across traits. MiXeR [6] was used in conjunction with causal mixture models to refine the estimates of genetic overlap between these traits, leveraging the methodology detailed in prior studies and available at https://github.com/precimed/mixer.

Pleio-informed Conditional False Discovery Rate Method

Conditional False Discovery Rate (condFDR)

The conditional Q-Q and fold-enrichment plots correlate with the true discovery rate (TDR), expressed as TDR = 1 - False discovery rate (FDR) [7]. The FDR is calculated for the specific p-value threshold as:

$$FDR\,(p) = F(p)\pi_0 F_0(p) \qquad \text{(S1)}$$

Here, π_0 indicates the proportion of null SNPs, with F_0 representing the cumulative distribution function (cdf) for null SNPs, and F including both null and non-null SNPs (8). When the null hypothesis is true, F_0 corresponds to the cdf of the uniform distribution across [0,1], simplifying the FDR:

$$FDR\ (p) = F(p)\pi_0 p \qquad \text{(S2)}$$

The empirical cumulative distribution factor cdf $q = Np/N$ estimates F. Therefore, substituting q *for* F in Equation [2] results in:

$$\text{Estimated FDR}\ (p) = \pi_0\, p/q \qquad \text{(S3)}$$

recognized as the overestimate of the FDR [9]. Further bias is introduced if π_0 is replaced with unity:

$$q* = p/q \qquad \text{(S4)}$$

the ratio of nominal against empirical quantiles, designated as $q*$ in Q-Q plots, allowing the direct estimation of FDR from the plot:

$$-\log 10\ (q*) = \log 10\ (q) - \log 10\ (p) \qquad \text{(S5)}$$

where a larger deviation from the $x = y$ line in the plots indicates a lower FDR.

Conditional Q-Q Plots

Conditional Q-Q plots are effective tools in large-scale studies such as genome-wide association studies, providing true associations by analyzing summary statistics distributions [7, 10]. The Q-Q plots are commonly used to illustrate the statistical association enrichment compared to what would be predicted from the global null hypothesis. These plots compare nominal p-values originating from the GWAS dataset versus empirical distribution. If there are no true associations, nominal p-values against the empirical distribution will form a straight line on Q-Q plots. This expected line represents the theoretical p-values distributions, uniformly raging between (0,1) under the global null hypothesis. Therefore, deviations from this line in Q-Q plots visually identify about the enrichment or depletion of statistical associations [7, 10].

In GWAS analysis, it is common to generate plots where the -log10 p-values versus -log10 q-values were plotted, with q calculated as (1 – cdf (p)). This visual representation is employed to underscore the tail probabilities in both the theoretical and empirical distributions of associations linked to two associated phenotypes. A deviation towards the left from the predicted null line suggests higher tail probabilities within the z-scores. This leftward shift termed 'enrichment' presents an abundance of lower p-values, suggesting a genetic association with primary and secondary traits. Conditional Q-Q plots are generated by partitioning Single Nucleotide Polymorphisms (SNPs) into subsets according to an auxiliary variable associated with each SNP, with separate Q-Q plots generated for each distinct group. If variations in the auxiliary measure capture SNP enrichment, an increase in this measure will result in leftward deflection of these plots.

We created conditional Q-Q plots by calculating empirical quantiles across all SNPs and within groups based on their nominal p-values associated with conditional traits and vice versa. The empirical cumulative distribution (cdf) of nominal p-values was calculated across all SNPs and for SNPs reaching significance thresholds at $-\log10(p) \geq 1$, 2, and 3 (representing p-values less than 1, 0.1,0.01, 0.001 respectively) for particular phenotypes [10-14]. In these plots, SNPs with nominal –log10 (p) values less than 7.3 ($p > 5x10^{-8}$) were investigated to uncover polygenic signals that did not reach typical GWAS significant criteria. Conditional Q-Q plots were generated by performing random pruning to reduce false enrichment, averaging over 500 iterations. In each iteration, a single SNP per LD block ($r^2 > 0.1$) was selected at random, and the respective p-values were used to calculate empirical cumulative distribution functions (cdfs).

Fold Enrichment Plots

We employed fold enrichment plots for visual inspection of the relationship between primary and conditional traits. Enrichment, indicating deviations from the expected null level, was assessed based on the conditional trait's associated p-values, which defined specific thresholds for trait association. To

create these plots, we first estimated the cdf of the p-values of the primary trait across all SNPs, followed by computing the cdf for each SNP stratum using conditional trait p-values. The fold enrichment was then calculated as the ratio of cdf for each stratum to the overall cdf, with plots illustrating the nominal -log10(p-value) for the primary trait along the x-axis, with fold enrichment on the y-axis, emphasizing the polygenic SNPs effect below the standard genome-wide significance level (-log10 (p) < 7.30, or p > 5×10^{-8}).

Detection of Genetic Variants by Using Conditional and Conjunctional FDR

To detect genetic variants the cond/conjFDR approach was utilized for the detection of genetic variants [10, 11]. The standard false discovery rate indicates the probability that an SNP is null, considering that its p-value is equal to or lower than the observed one. The conditional FDR (condFDR; an extension of standard FDR) integrates data from GWAS datasets of primary and secondary traits for adjusting the significance level. The condFDR estimates the probability that an SNP is null for the first trait, considering that the p values for both traits are low or lower than observed ones. The SNPs are ranked similarly by standard FDR or p-value; however, condFDR reorders them differently in the presence of a genetic association between traits of interest. The code utilized for this analysis can be retrieved from https://github.com/precimed/pleiofdr [10-12]. For this study, we utilized a 1000 Genomes phase 3 dataset as a reference panel that includes participants from European ancestry [15].

The conjFDR [10] (an extension of condFDR), is the posterior probability that an SNP shows no association with either one or both phenotypes, where its P-values for both traits are as small or smaller than the observed one. To obtain a conservative conjFDR estimate, the highest condFDR for an SNP is derived by conducting the condFDR for both traits with their roles reversed [16]. Given the potential influence of complex correlations within regions characterized by intricate linkage disequilibrium (LD) [17].We chose to exclude single nucleotide polymorphisms (SNPs) located within the extended major histocompatibility complex (MHC) region found on chromosome 6 (hg19: 25,119,106-33,854,733) and on chromosome 8p23.1 (positions in hg19: 7242715–12483982). Additionally, SNPs exhibiting LD with a correlation coefficient (r2) greater than 0.1 were also excluded before applying FDR models. Furthermore, we constructed a Manhattan plot for three or four traits that shared common variants at conjFDR < 0.05. Genomic control was applied to correct inflation in the p-values [10].

Genetic Correlation

We applied a statistical method known as linkage disequilibrium score regression (LDSC) [24] for the estimation of genetic heritability and genetic correlation among LON, MDD, and sleep traits [18]. This analysis utilized the Python-based toolkit accessible from the repository (https://github.com/bulik/ldsc). The methodology details are explained thoroughly in this package (https://github.com/bulik/ldsc/wiki/Heritability-and-Genetic-Correlation).

Genomic Loci Definition, Functional Mapping and Annotation

The independent genomic loci were determined by following the FUMA protocol [19] (http://fuma.ctglab.nl/). The candidate SNPs within loci having conjFDR values less than 0.10 and LD r2 of at least 0.6 with independent significant SNPs that were functionally annotated by multiple tools in FUMA. Combined Annotation Dependent Depletion (CADD) score [20] annotated SNPs to predict deleteriousness on protein structure and function, CADD score above 12.37 shows high deleteriousness. *RegulomeDB* (RDB) scores [21] ranging from 1a to 7, estimating SNPs for regulatory potential functions using eQTL and chromatin marks information [22], with lower scores indicating transcription or regulatory influences on SNP locus. The chromatin states categorize genomic accessibility with every 200 bp into 15 different states. This prediction is generated by ChromHMM, a hidden Markov model using 5 chromatin marks across 127 epigenomes [22]. The default parameters of FUMA were used for this analysis.

We employed three gene mapping approaches to connect independent SNPs within shared loci to brain-expressed genes [19]. First, positional mapping assigns SNPs to loci (within a 10kb range) according to their physical proximity to all genes. Second, eQTL mapping aligns SNPs with genes through the expression of allelic variation as a reference. Third, chromatin interaction mapping aligns the SNPs to genes through 3-dimensional DNA-DNA interactions. We integrated brain tissue eQTL databases in FUMA. All analyses undergo multiple comparisons by using Bonferroni correction.

Supplementary Results

Allelic Effect Directions

The directions of allelic effect on the traits of interest were calculated by analyzing z-scores of lead SNPs. We found the same effect direction in 58/62 loci (93.5%) of loneliness and major depression, 16/56 loci (28.5%) of loneliness and insomnia, 18/54 loci (33.3%) of loneliness and chronotype, 15/62 loci (24%) of loneliness and sleep duration, 7/19 loci (36.8%) of major depression and insomnia, 7/17 loci (41%) of major depression and chronotype, and 7/15 loci (46.6%) of major depression and sleep duration.

Functional Annotation of Shared Loci

Using FUMA, we conducted functional annotations of all the SNPs having linkage disequilibrium (LD) ($r^2 \geq 0.6$) at cond/conjFDR < 0.10 within the genomic risk loci related to LON, MDD, and sleep traits with one of the independent SNPs. The distribution of functional consequences indicates variations across different SNP sets. Among all the SNPs associated with both LON and MDD, 63.3% were in intronic regions, 30.1% were in intergenic regions, and 1.5% were in exonic regions. In the case of SNPs associated with both LON and INS, 63.5% were in intronic regions, 29.1% were found in intergenic regions, and 1.0% were in exonic regions. Similarly, for SNPs jointly associated with LON and CHR, 73% were intronic, 20.2% were intergenic, and 2.5% were exonic. In the case of SNPs associated with both LON and SD, 76.7% were intronic, 16.8% were intergenic, and 21.2% were in exonic regions. In addition, the percentages for SNPs associated with MDD and sleep traits including insomnia (INS), chronotype (CHR), and sleep duration (SD) also varied across intronic, intergenic and exonic regions. Specifically, among SNPs jointly associated with MDD and INS, 41.1% were intronic, 52.7% were intergenic, and 1.6% were in exonic regions. Similarly, for SNPs jointly associated with MDD and CHR, 63.5% were intronic, 29.1% were intergenic, and 1.0% were exonic. In the case of SNPs found in both MDD and SD, most SNPs (58.5%) were present in intronic regions, with 31.8% in intergenic and 3.0% in exonic regions (Manuscript Figure 6, Fig. S5, Supplemetary Material File 2: Supplementary Table S62).

Additional findings indicated that 3.7% (n = 43) of the identified candidate SNPs associated with both LON and MDD exhibited CADD scores exceeding 12.37, indicating significant deleteriousness; four of them had 1f RegulomeDB scores potentially affecting transcription factor binding (Supplementary Material File 2: Table S23). In the case of candidate SNP jointly associated with LON and INS, 3.4% (n = 42) displayed considerable deleteriousness; one of these SNPs, "rs10865954", had a CADD score <15.24 and 1f RegulomeDB (Supplementary Material File 2: Table S24). For SNPs jointly associated with LON and CHR, 3.5% (n = 100) showed high deleteriousness, and five of them had low RegulomeDB scores of 1f (Supplementary Material File 2: Table S25). Similarly, among SNPs linked to LON and SD, 3.6% (n = 113), showed a similar pattern of high deleteriousness, and seven of them had 1f RegulomeDB scores (Supplementary Material File 2: Table S26). For candidate SNPs jointly

associated with MDD and INS, 9.6% (n=18) showed high CADD scores greater than 12.37, indicating significant deleteriousness (Supplementary Material File 2: Table S27). In the case of SNPs linked with MDD and CHR, 5.6% (n= 6) showed high deleteriousness (Supplementary Material File 2: Table S28). Similarly, 6.3% (n=29) of the candidate SNPs associated with both MDD and SD showed high CADD scores (Supplementary Material File 2: Table S29).

Gene Mapping, Gene-Set, and Pathway Analysis Results

We used three different gene mapping approaches in FUMA to assign independent single nucleotide polymorphism (SNPs) within shared loci to brain-expressed genes. Among these independent SNPs jointly associated with LON and MDD, 24 genes were mapped using positional mapping, 105 genes through eQTL mapping, and 86 genes mapped via chromatin interaction mapping strategy. In the case of LON and INS, 62 genes were mapped through positional, 119 with eQTL and 119 genes were mapped through chromatin interaction mapping. For SNPs shared with LON and CHR, the positional mapping strategy mapped the SNPs to 91 genes, and eQTL aligned the SNPs to 221 genes, whereas 151 genes aligned through chromatin interaction mapping. Among SNPs shared with LON and SD, 86 genes were aligned through positional, 209 genes through eQTL, and 178 genes through the chromatin interaction mapping approach. In addition, among SNPs shared with MDD and INS, 16 genes aligned through positional mapping, 6 genes through eQTL, and 34 genes through chromatin interaction mapping. In the case of MDD and CHR, positional mapping implicated SNPs with 10 genes, eQTL-linked SNPs to 29 genes, and 9 genes were mapped through chromatin interaction mapping. Moreover, for MDD and SD, 40 genes were mapped through positional mapping, 75 through eQTL, and 95 genes aligned with SNPs through chromatin interaction mapping. Among these, 120 genes were aligned using at least two out of three different gene mapping approaches (Supplementary File 2: Table 2).

Using gene set and pathway analysis, 51 significant Gene Ontology (GO) terms were identified [22, 23] for LON, MDD, and sleep-related phenotypes associated with several biological, molecular, and cellular components including "structural constituent of chromatin", "protein heterodimerization activity", "nucleosome organization", "DNA packaging complex", "odorant binding" and "olfactory receptor activity" etc. (Supplementary Material File 2: Table S54). Gene set pathway analysis also discovered several pathways after excluding MHC regions (Supplementary Material File 2: Tables S55–S61). The shared pathways were "Kegg-systemic_lupus_erythematosus", "signaling by wnt", "olfactory signaling pathway", "signaling by notch", "signaling by nuclear receptors", "signaling by rho_gtpases_miro_gtpases_and_rhobtb3", "ESR_mediated_signaling" etc.

Supplementary Tables

Table S2. Novel Risk Variants Associated with LON, MDD, and Sleep Traits at conjFDR < 0.05.

Phenotypes	Distinct Genomic Loci at ConjFDR < 0.05	Novel variants for LON and MDD
**LON丨MDD**	62	53
**LON丨INS**	56	52
**LON丨CHR**	54	48
**LON丨SD**	62	58
**MDD丨CHR**	17	12
**MDD丨INS**	19	03
**MDD丨SD**	15	07

Note: LON: Loneliness, MDD: Major depressive disorder, INS: Insomnia, CHR: Chronotype, SD: Sleep duration.

Table S3: Common Risk Factors Shared between LON, MDD, and Sleep Traits at ConjFDR < 0.05.

CHR	LEAD_SNP	MinBP	MaxBP	A1/A2	nearestGene	func	Novel in LON	Shared with
5	rs30266	103671867	104089064	A/G	*RP11-6N13.1*	ncRNA_intronic	NO	MDD, CHR, INS
18	rs10503002	53057188	53164693	T/C	*TCF4*	intronic	YES	MDD, CHR, INS
7	rs71573104	114118112	114249522	G/A	*FOXP2*	intronic	YES	MDD, SD
11	rs7939345	47962298	48966394	T/G	*PTPRJ*	intergenic	YES	MDD, SD
14	rs7141014	98603851	98670849	C/T	*RP11-61O1.1*	ncRNA_intronic	YES	MDD, INS

Abbreviations: Loneliness (LON), Major Depressive Disorder (MDD), Insomnia (INS), Chronotype (CHR), Sleep Duration (SD), Single-Nucleotide Polymorphism (SNP), Allele 1/Allele 2 (A1/A2), MinBP (Minimum base position), Function (func), MaxBP (Maximum base position). The base pair positions were determined using the GRCh37/Hg19 genomic build.

Table S4. LAVA – Number of Positively and Negatively Correlated Genomic Regions between Loneliness (LON), Major Depressive Disorder (MDD), Chronotype (CHR), and Sleep Duration (SD).

Genomic regions in the univariate LAVA		Genomic regions in the Bivariate LAVA			
**LON**	**Secondary Phenotype**	**Total**	**Significant (P<0.05)**	**Concordant**	**Discordant**
2111	MDD: 2085	1639	10	10	0
	CHR: 2330	1813	2	0	2
	INS: 2458	1897	3	3	0
	SD: 2228	1744	4	0	4

Supplementary Figures

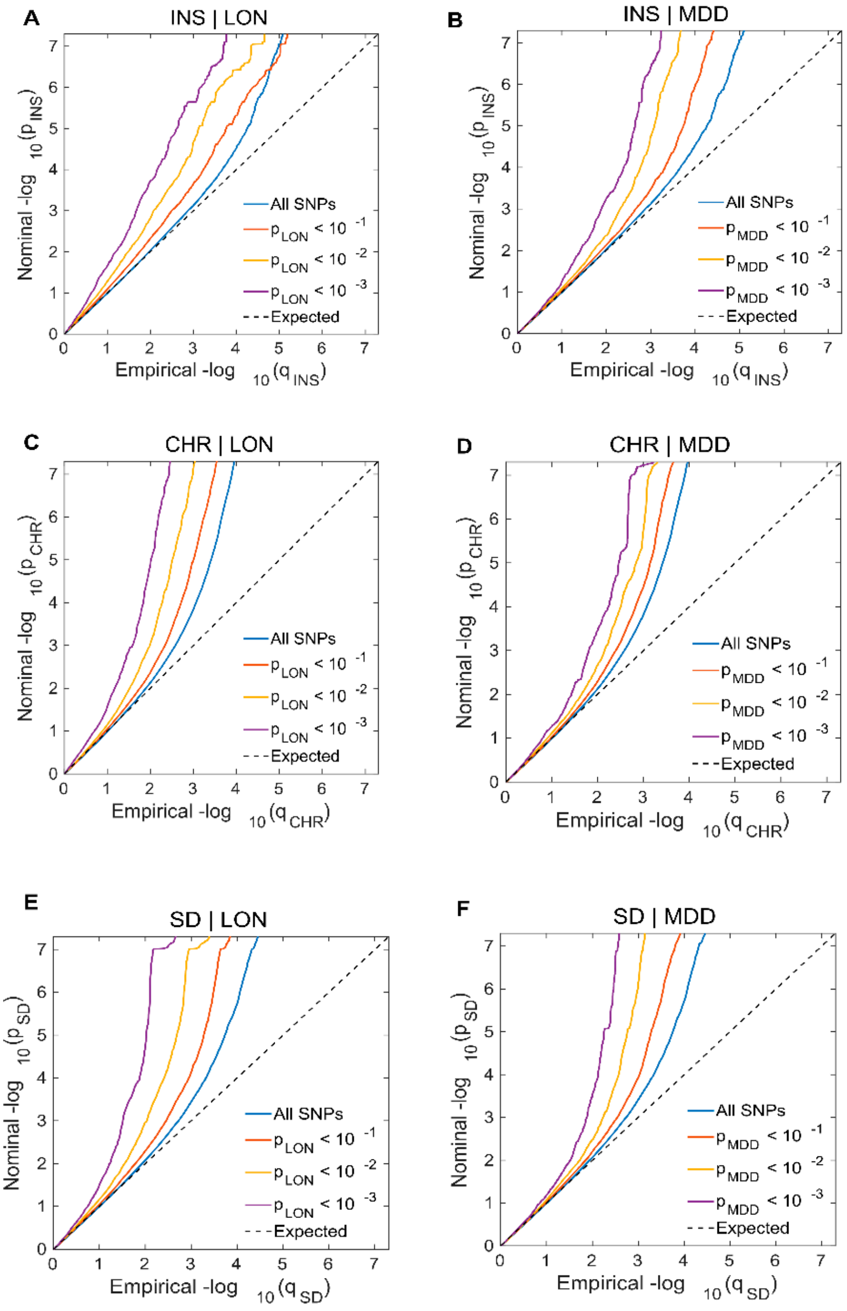

Figure S1. Genetic overlap of sleep traits with LON and MDD. **(A-F)** Conditional Q-Q plots display polygenic enrichment among INS, CHR, and SD conditioned on LON and MDD by plotting the nominal p-values against the inflation-adjusted empirical -log10(p) values for the phenotypes under investigation, following standard GWAS criteria of $p < 5e\text{-}8$. Significant association with the conditional trait is represented by -log10 p-values cut-offs ($p < 0.10, 0.01, 0.001$). The dotted lines specify the null hypothesis, and the blue lines indicate all the SNPs.

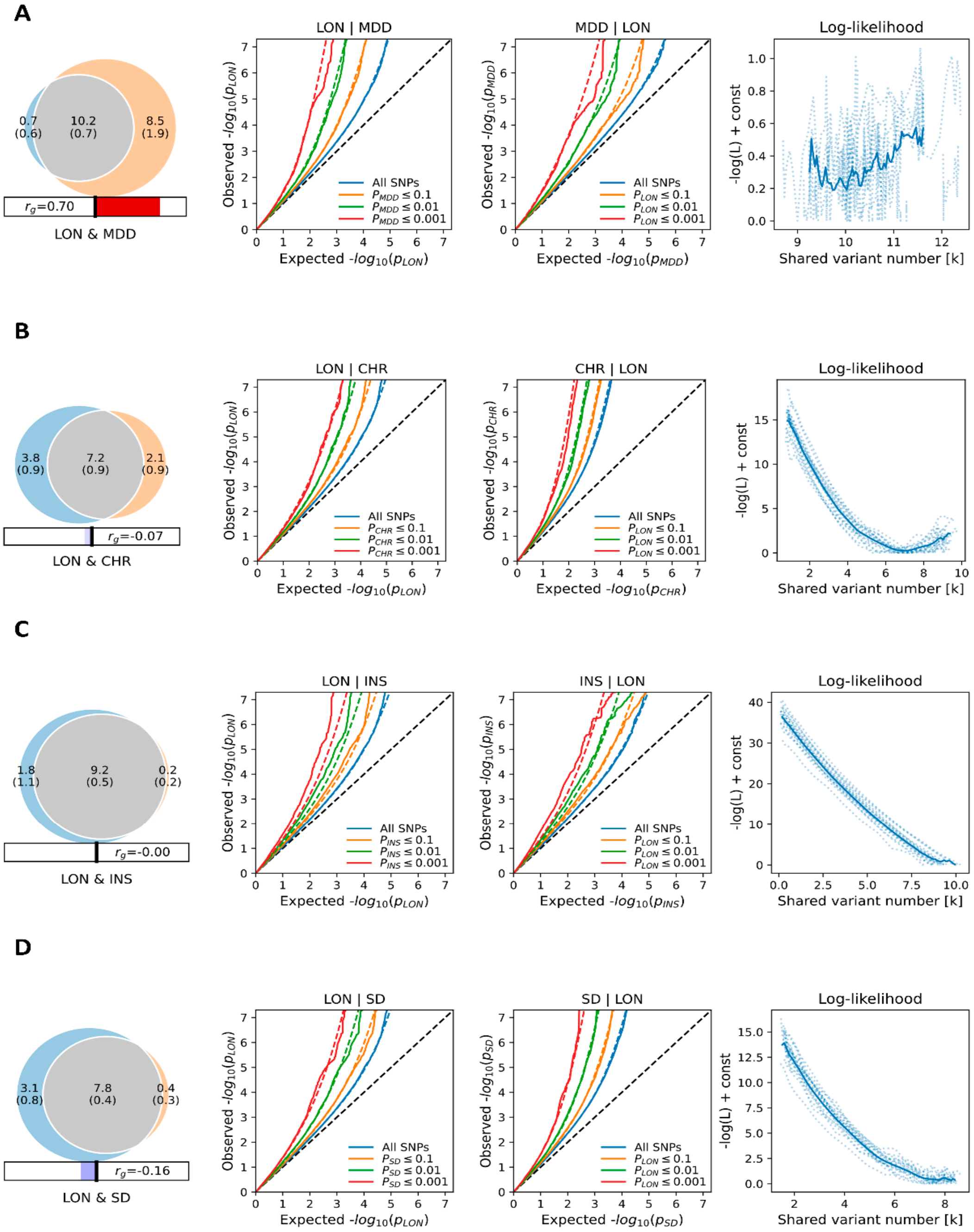

Figure S2. MiXeR figures show polygenicity among (**A**) LON and MDD, (**B**) LON and CHR, (**C**) LON and INS, and (**D**) LON and SD. *Left panels:* Venn diagrams showed shared (gray color) and unique variants among LON (blue color) and secondary phenotypes (orange color), accounting for 90% of SNP heritability in each trait. The rg refers to the genome-wide significant correlation (directional scale: positive, red; negative, blue). The size of the circle corresponds to the polygenicity of each trait, with large circles representing the higher polygenicity. *Middle panels:* Conditional Q-Q plots of observed versus expected $-\log_{10}$ p-values in the primary phenotype as a function of significance of association with the secondary phenotype at the level of $p < 0.1$ (orange lines), 0.01 (green lines), 0.001 (red lines). The blue lines represent all single-nucleotide polymorphisms (SNPs). The blue, orange, green and red dotted lines indicate the model prediction for each stratum. *Right panels:* MiXeR model negative log-likelihood plots fit as a function of π parameter. The remaining parameters are restricted to their respective fitted values.

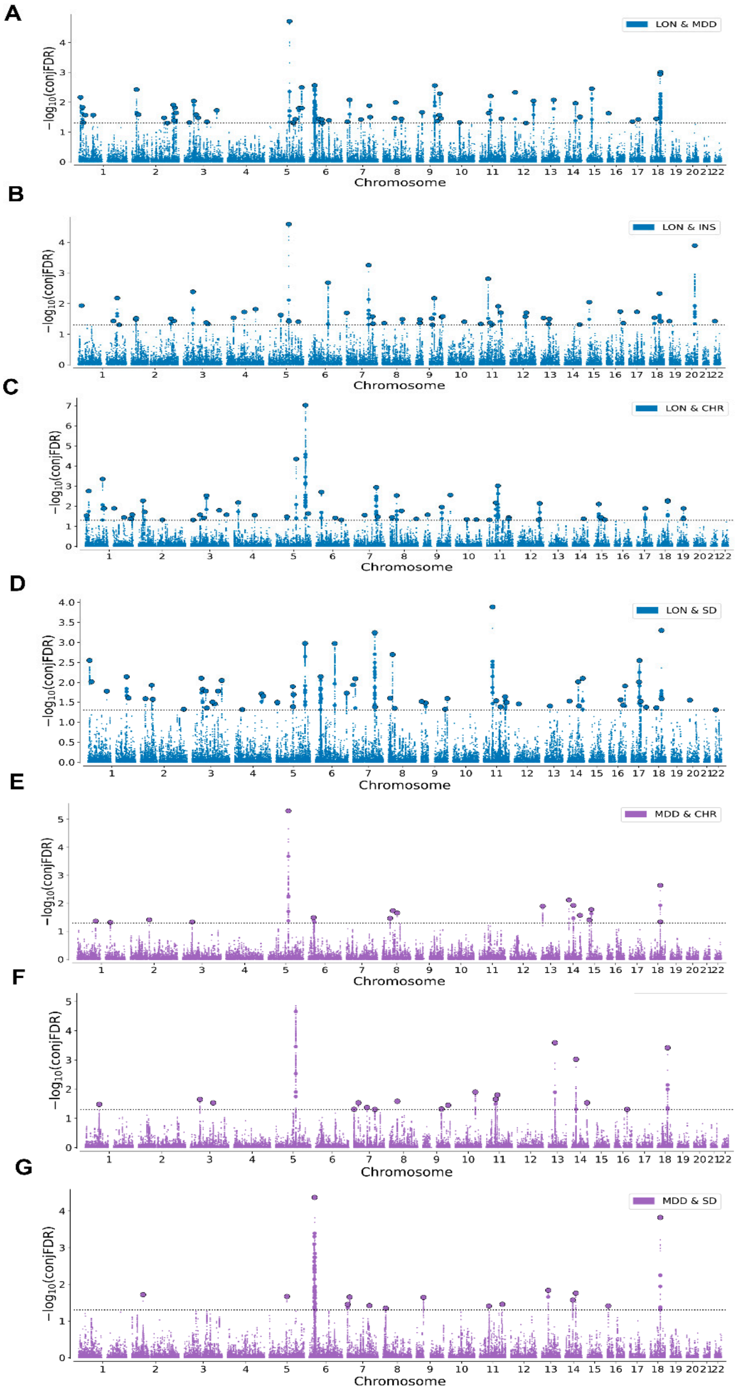

Figure S3. (**A-G**) Manhattan plots for LON, MDD, CHR, and INS at conjFDR < 0.05. This figure presents Manhattan plots in which the y-axis illustrates -$\log_{10}$ transformed conjFDR values for the SNPs, whereas the x-axis shows chromosomal positions. Black circles surrounding the larger data points illustrate the significant SNPs found in each LD block. The dashed horizontal line denotes the significant threshold to identify shared and common associations when conjFDR is less than 0.05.

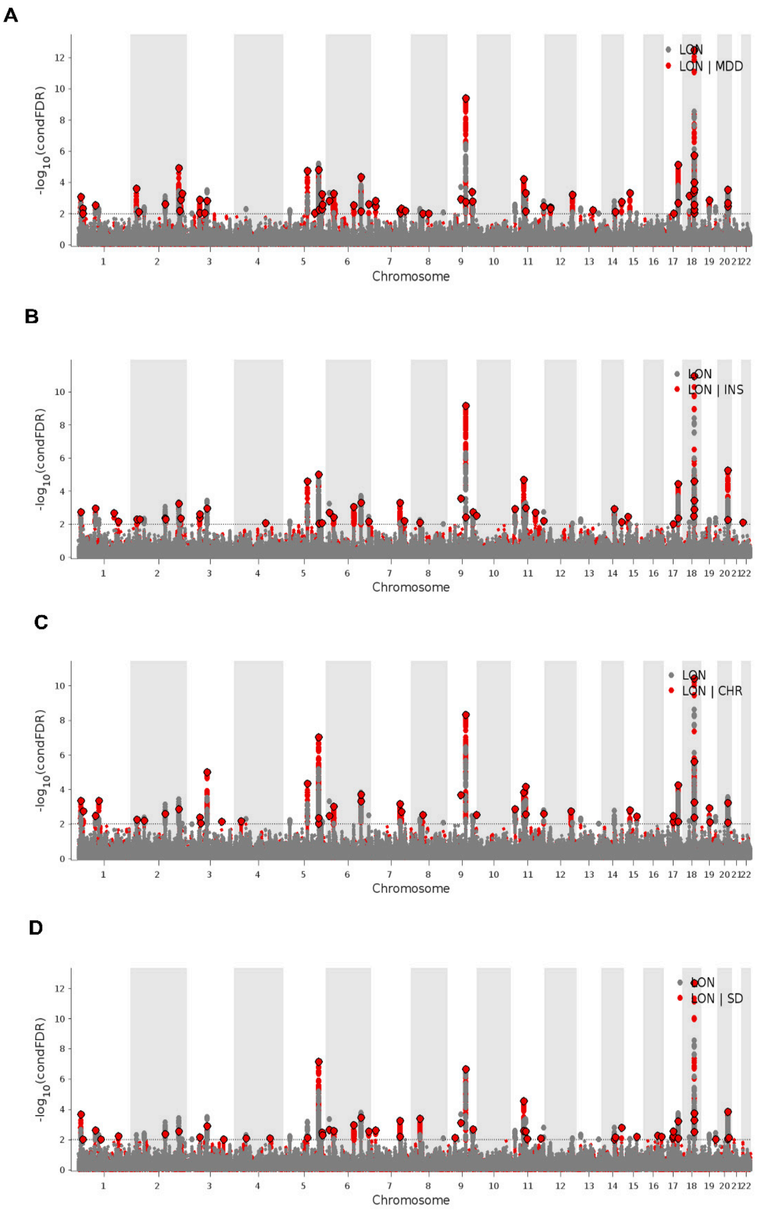

Figure S4. Specific genomic risk loci associated with primary trait at condFDR < 0.01, based on their association with secondary trait. Manhattan plots for (**A**) LON and MDD, (**B**) LON and INS, (**C**) LON and CHR, and (**D**) LON and SD. Black circles surrounding the larger data points represent the significant SNPs within each LD block.

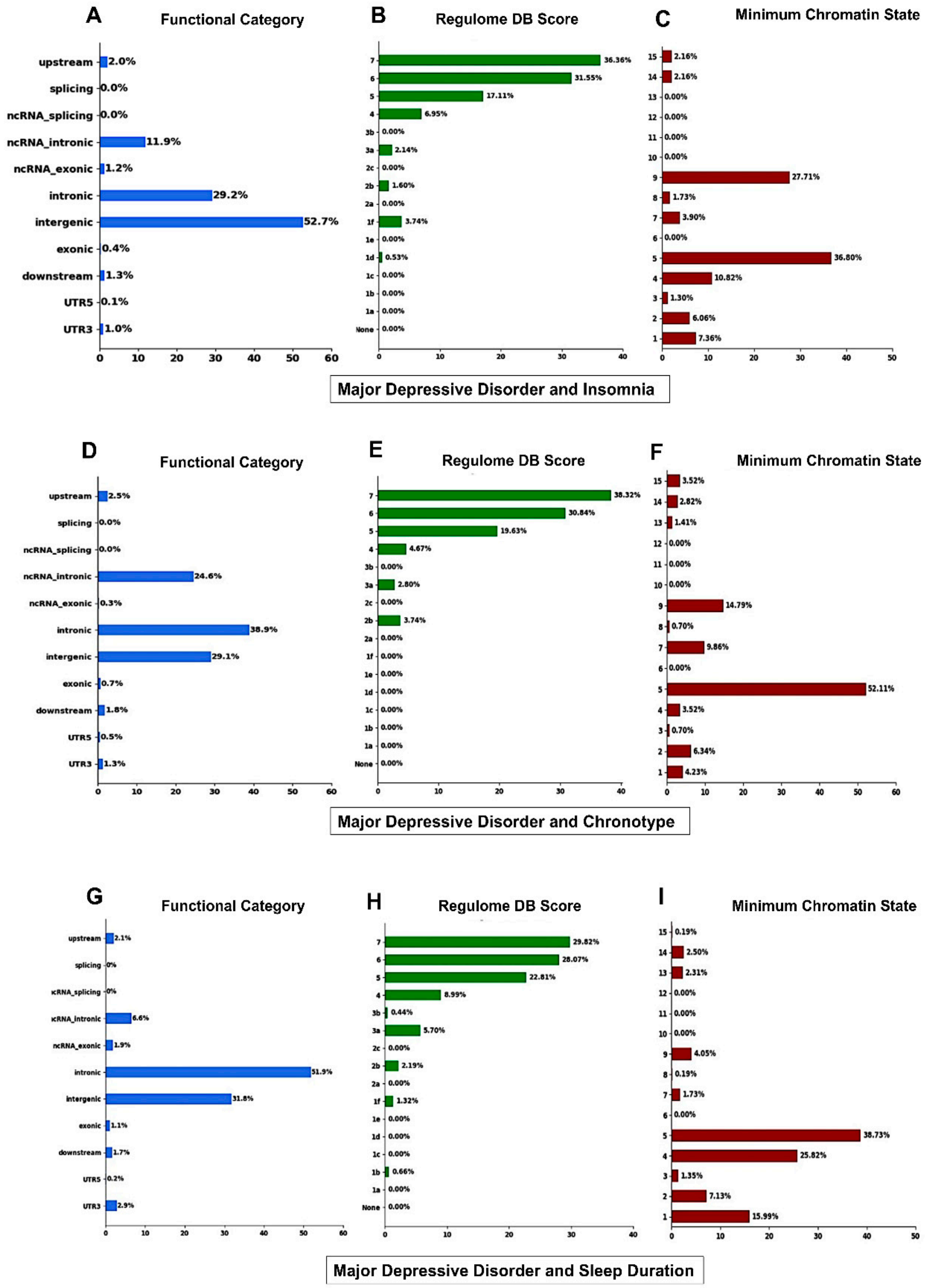

Figure S5. (A-H) The functional annotation distribution, RegulomeDB Score, and Chromatin interaction mapping of all SNPs within the common genetic risk variants between major depression and sleep traits at conjFDR < 0.10.

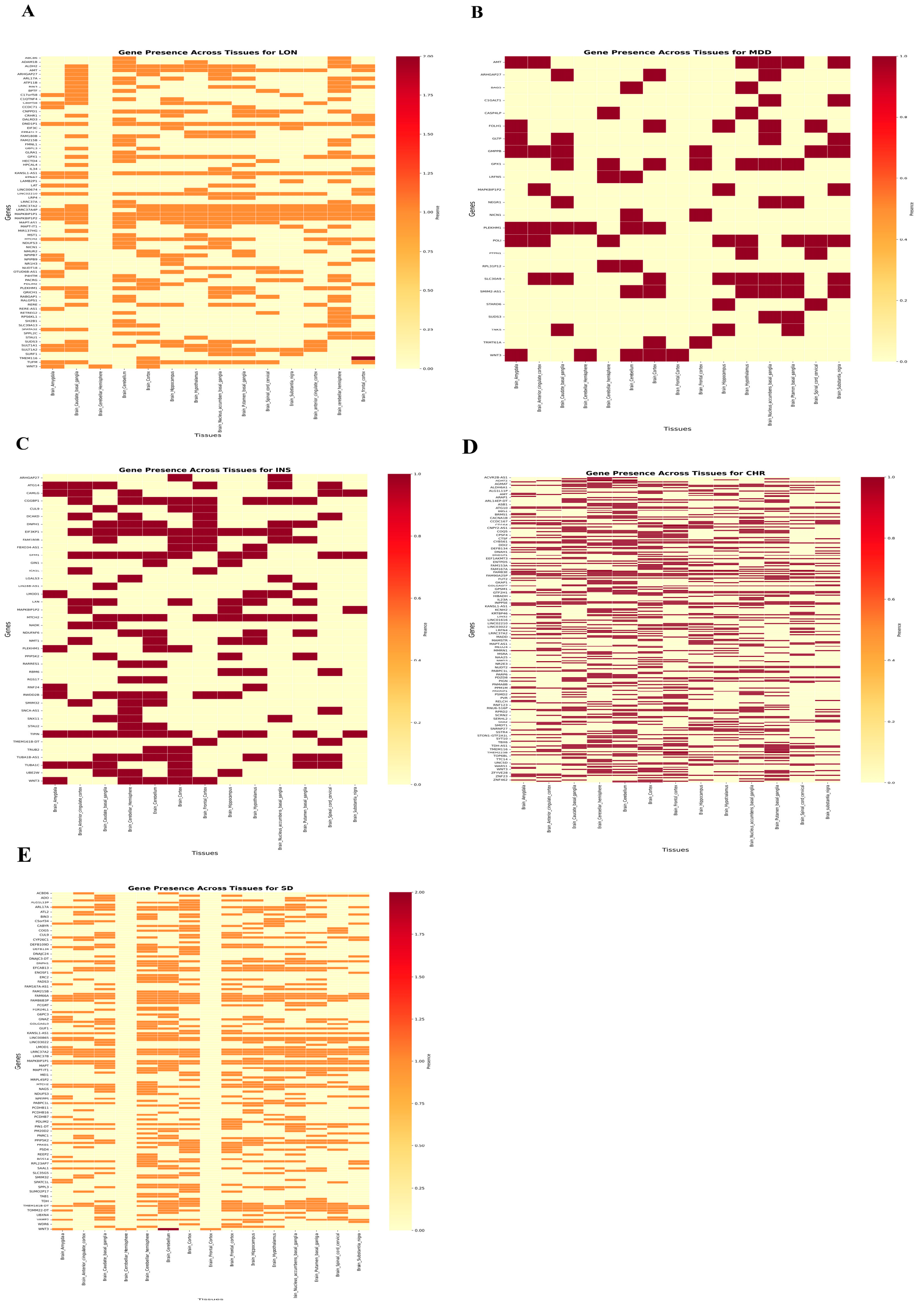

Figure S6: (A-E) Heatmaps for LON, MDD, INS, CHR, and SD showing all TWAS significant genes. Columns represent brain regions, and rows represent TWAS-significant genes for each trait. Color scale indicates effect direction and magnitude.

References

1. Day FR, Ong KK, Perry JR. Elucidating the genetic basis of social interaction and isolation. Nature communications. 2018;9(1):2457.
2. Wray NR, Ripke S, Mattheisen M, Trzaskowski M, Byrne EM, Abdellaoui A, et al. Genome-wide association analyses identify 44 risk variants and refine the genetic architecture of major depression. Nature genetics. 2018;50(5):668-81.
3. Watanabe K, Jansen PR, Savage JE, Nandakumar P, Wang X, Hinds DA, et al. Genome-wide meta-analysis of insomnia prioritizes genes associated with metabolic and psychiatric pathways. Nature genetics. 2022;54(8):1125-32.
4. Jones SE, Lane JM, Wood AR, van Hees VT, Tyrrell J, Beaumont RN, et al. Genome-wide association analyses of chronotype in 697,828 individuals provides insights into circadian rhythms. Nature communications. 2019;10(1):343.
5. Dashti HS, Jones SE, Wood AR, Lane JM, Van Hees VT, Wang H, et al. Genome-wide association study identifies genetic loci for self-reported habitual sleep duration supported by accelerometer-derived estimates. Nature communications. 2019;10(1):1100.
6. Frei O, Holland D, Smeland OB, Shadrin AA, Fan CC, Maeland S, et al. Bivariate causal mixture model quantifies polygenic overlap between complex traits beyond genetic correlation. Nature communications. 2019;10(1):2417.
7. Benjamini Y, Hochberg Y. Controlling the false discovery rate: a practical and powerful approach to multiple testing. Journal of the Royal statistical society: series B (Methodological). 1995;57(1):289-300.
8. Efron B. Size, power and false discovery rates. 2007.
9. Purcell S, Neale B, Todd-Brown K, Thomas L, Ferreira MA, Bender D, et al. PLINK: a tool set for whole-genome association and population-based linkage analyses. The American journal of human genetics. 2007;81(3):559-75.
10. Andreassen OA, Djurovic S, Thompson WK, Schork AJ, Kendler KS, O'Donovan MC, et al. Improved detection of common variants associated with schizophrenia by leveraging pleiotropy with cardiovascular-disease risk factors. The American Journal of Human Genetics. 2013;92(2):197-209.
11. Andreassen OA, Thompson WK, Dale AM. Boosting the power of schizophrenia genetics by leveraging new statistical tools. Schizophrenia bulletin. 2014;40(1):13-7.
12. Smeland OB, Frei O, Shadrin A, O'Connell K, Fan C-C, Bahrami S, et al. Discovery of shared genomic loci using the conditional false discovery rate approach. Human genetics. 2020;139(1):85-94.
13. Schork AJ, Wang Y, Thompson WK, Dale AM, Andreassen OA. New statistical approaches exploit the polygenic architecture of schizophrenia—implications for the underlying neurobiology. Current opinion in neurobiology. 2016;36:89-98.
14. Liu JZ, Hov JR, Folseraas T, Ellinghaus E, Rushbrook SM, Doncheva NT, et al. Dense genotyping of immune-related disease regions identifies nine new risk loci for primary sclerosing cholangitis. Nature genetics. 2013;45(6):670-5.
15. Consortium GP. A global reference for human genetic variation. Nature. 2015;526(7571):68.
16. Nichols T, Brett M, Andersson J, Wager T, Poline J-B. Valid conjunction inference with the minimum statistic. Neuroimage. 2005;25(3):653-60.
17. Schwartzman A, Lin X. The effect of correlation in false discovery rate estimation. Biometrika. 2011;98(1):199-214.
18. Bulik-Sullivan BK, Loh P-R, Finucane HK, Ripke S, Yang J, Consortium SWGotPG, et al. LD Score regression distinguishes confounding from polygenicity in genome-wide association studies. Nature genetics. 2015;47(3):291-5.
19. Watanabe K, Taskesen E, Van Bochoven A, Posthuma D. Functional mapping and annotation of genetic associations with FUMA. Nature communications. 2017;8(1):1826.
20. Kircher M, Witten DM, Jain P, O'roak BJ, Cooper GM, Shendure J. A general framework for estimating the relative pathogenicity of human genetic variants. Nature genetics. 2014;46(3):310-5.

21. Boyle AP, Hong EL, Hariharan M, Cheng Y, Schaub MA, Kasowski M, et al. Annotation of functional variation in personal genomes using RegulomeDB. Genome research. 2012;22(9):1790-7.
22. Kundaje A, Meuleman W, Ernst J, Bilenky M, Yen A, Heravi-Moussavi A, et al. Integrative analysis of 111 reference human epigenomes. Nature. 2015;518(7539):317-30.

References [9,13,14,16,17] are cited in the Supplementary Materials.
